# Activating *PAX* gene family paralogs to complement *PAX5* leukemia driver mutations

**DOI:** 10.1371/journal.pgen.1007642

**Published:** 2018-09-14

**Authors:** Matthew R. Hart, Donovan J. Anderson, Christopher C. Porter, Tobias Neff, Michael Levin, Marshall S. Horwitz

**Affiliations:** 1 Allen Discovery Center and Department of Pathology, University of Washington School of Medicine, Seattle, Washington, United States of America; 2 University of Colorado School of Medicine, Aurora, Colorado, United States of America; 3 Allen Discovery Center and Biology Department, Tufts University, Medford, Massachusetts, United States of America; Massachusetts General Hospital Cancer Center, UNITED STATES

## Abstract

*PAX5*, one of nine members of the mammalian paired box (*PAX*) family of transcription factors, plays an important role in B cell development. Approximately one-third of individuals with pre-B acute lymphoblastic leukemia (ALL) acquire heterozygous inactivating mutations of *PAX5* in malignant cells, and heterozygous germline loss-of-function *PAX5* mutations cause autosomal dominant predisposition to ALL. At least in mice, *Pax5* is required for pre-B cell maturation, and leukemic remission occurs when *Pax5* expression is restored in a *Pax5*-deficient mouse model of ALL. Together, these observations indicate that *PAX5* deficiency reversibly drives leukemogenesis. *PAX5* and its two most closely related paralogs, *PAX2* and *PAX8*, which are not mutated in ALL, exhibit overlapping expression and function redundantly during embryonic development. However, *PAX5* alone is expressed in lymphocytes, while *PAX2* and *PAX8* are predominantly specific to kidney and thyroid, respectively. We show that forced expression of *PAX2* or *PAX8* complements *PAX5* loss-of-function mutation in ALL cells as determined by modulation of *PAX5* target genes, restoration of immunophenotypic and morphological differentiation, and, ultimately, reduction of replicative potential. Activation of *PAX5* paralogs, *PAX2* or *PAX8*, ordinarily silenced in lymphocytes, may therefore represent a novel approach for treating *PAX5*-deficient ALL. In pursuit of this strategy, we took advantage of the fact that, in kidney, *PAX2* is upregulated by extracellular hyperosmolarity. We found that hyperosmolarity, at potentially clinically achievable levels, transcriptionally activates endogenous *PAX2* in ALL cells via a mechanism dependent on NFAT5, a transcription factor coordinating response to hyperosmolarity. We also found that hyperosmolarity upregulates residual wild type *PAX5* expression in ALL cells and modulates gene expression, including in *PAX5*-mutant primary ALL cells. These findings specifically demonstrate that osmosensing pathways may represent a new therapeutic target for ALL and more broadly point toward the possibility of using gene paralogs to rescue mutations driving cancer and other diseases.

## Author summary

Mutations inactivating *PAX5* disrupt B cell differentiation and occur frequently in ALL. Others have previously shown that restoring *PAX5* expression normalizes B cell differentiation and leads to disease remission in a mouse model of ALL. We found that exogenous expression of *PAX5*’s intact and closely related gene family members, *PAX2* or *PAX8*, which are ordinarily silent in lymphocytes but expressed in kidney and other tissues, can substitute for *PAX5* and restore differentiation in ALL cells. A new approach for treating ALL might therefore be to discover ways to activate expression of *PAX2* or *PAX8* in leukemic cells. In the kidney, *PAX2* expression is activated by changes in extracellular osmolarity. We found that *PAX2* retains the capacity for osmotic activation in ALL cells and that wild type *PAX5* expression also increases when ALL cells are osmotically stressed. Adjustment of serum osmolarity—or treatment with drugs targeting pathways responding to osmotic stress—may offer a potential new avenue for ALL therapy by elevating expression of *PAX* gene family members. More generally, our studies point toward a novel strategy of recruiting paralogs to complement mutations in genes responsible for cancer and other diseases.

## Introduction

Pre-B acute lymphoblastic leukemia (ALL) is a common pediatric malignancy often successfully treated with chemotherapy [1]. Unfortunately, chemotherapy is not without side effects, including risk for secondary malignancies and other long-term complications [2]. Additionally, adolescents and adults fare less well, requiring greater reliance on allogeneic hematopoietic stem cell transplant [3]. While chimeric antigen receptor (CAR) T cell therapy for ALL [4] continues to advance, patients may benefit from additional therapeutic options.

As with other types of leukemia, pre-B ALL exhibits stage-specific hematopoietic developmental arrest, in this case, corresponding to hyperproliferation of immature B cell progenitors [5]. Treatment aimed at restoring differentiation capacity to leukemic cells has long been sought, but has proven elusive [6]. The only widely used form of differentiation therapy employs all-*trans* retinoic acid (ATRA), which has achieved remarkable success for the specific treatment of acute promyelocytic leukemia [7].

The transcription factor *PAX5* plays a central role in the origin of pre-B ALL as the single most common somatically mutated gene observed in the disease [8–10]. About one-third of patients acquire heterozygous *PAX5* mutations, with complete loss of both alleles rarely seen [9,11]. Deletions or other loss-of-function mutations are typical, but, less frequently, *PAX5* rearranges to form fusion genes with *ETV6* or other partners, generating dominant negative proteins [12]. Heterozygous germline *PAX5* loss-of-function mutation is also a cause of inherited predisposition to ALL [13,14]. In ALL cases defined by wild type *PAX5*, some acquire mutations in *EBF* or *E2A* (*TCF3*) [9], both of which are upstream activators of *PAX5* [5]. Functionally, *PAX5* activates B lymphoid-specific gene expression while repressing genes specifying alternative lineages, including T lymphocyte-promoting, *NOTCH1* [15]. As such, B lymphoid development in the bone marrow of *Pax5*-null mice arrests at the pre-B stage [16]. *Pax5* loss-of-function in conjunction with *Stat5* activation results in developmental blockage of the B cell transcriptional program and leukemic transformation in mice [17]. Importantly, forced re-expression of *PAX5* in *PAX5*-deficient ALL was recently shown to normalize growth and differentiation of leukemic cells in culture and clear circulating leukemic cells in a *Pax5*-deficient/*Stat5*-activated mouse model of ALL [18,19]. While cooperating mutations in additional genes arise during leukemogenesis [20], these findings, taken together, indicate that reduced *PAX5* activity reversibly drives the formation of pre-B ALL and represents an intriguing therapeutic target.

Nevertheless, modulating *PAX5* activity is likely to prove challenging. Transcription factors are generally regarded as “undruggable” [21]. Gene replacement therapy or genome editing [22] may ultimately prove too inefficient when dealing with large numbers of malignant cells. Moreover, targeting or even defining ALL leukemic stem cells for correction may be problematic, if not impossible [23]. However, in the case of genes that are members of paralogous gene families, such as *PAX5*, genetic redundancy may offer a feasible alternative.

**Fig 1:**
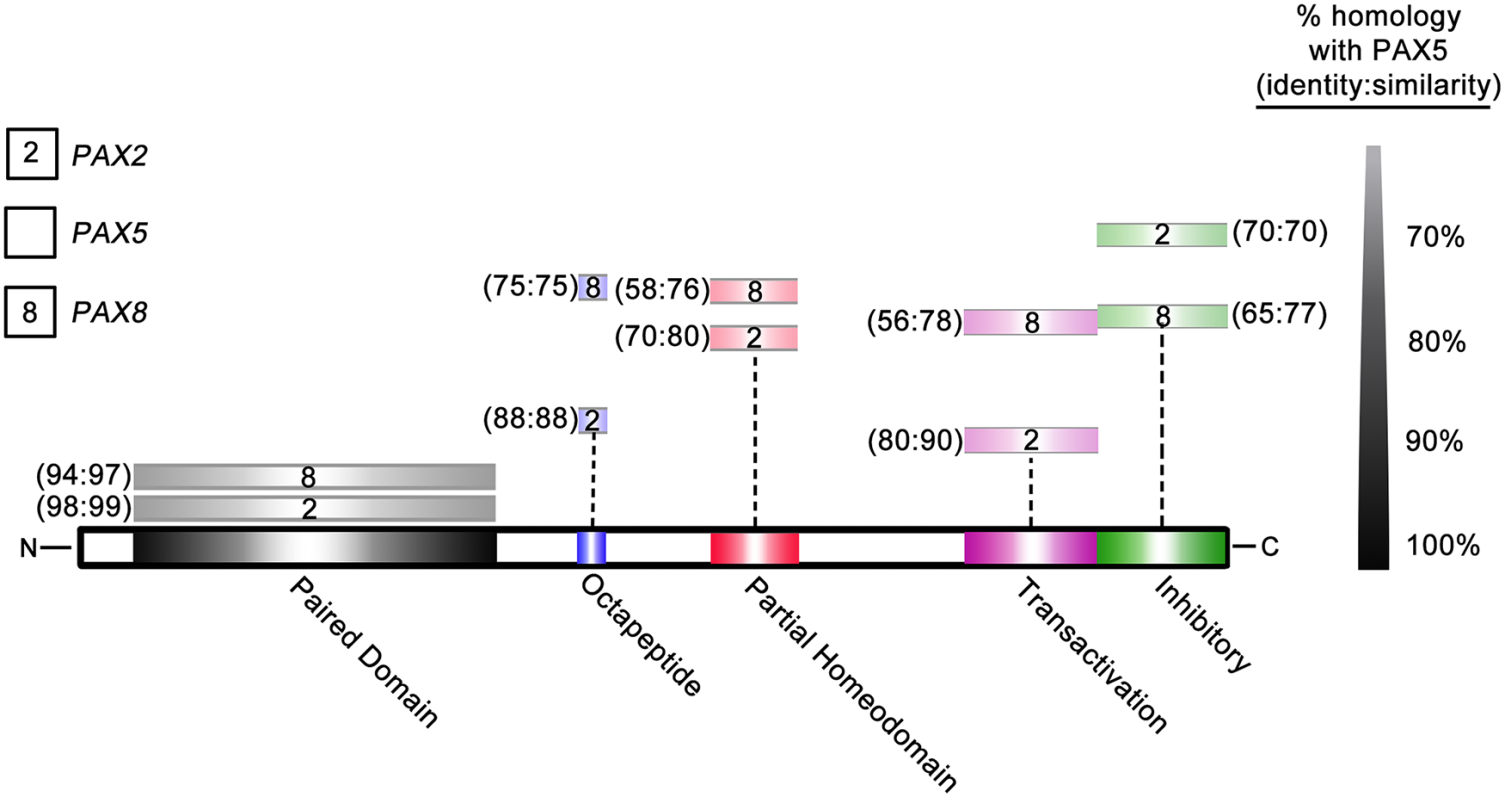
*PAX2*/*5*/*8* domains share high levels of homology. Schematic of full-length PAX5 protein. Equivalent domains of PAX2 and PAX8, indicated in key, are shown above. Distance from PAX5 represents level of homology to PAX5, scale at right. See also S1 Fig.

The mammalian *PAX* gene family consists of nine paralogs [24]. Divergence among its four subfamilies is largely non-coding, within *cis* regulatory regions, allowing for tissue specific expression among family members [25]. In particular, members of the *PAX2*/*5*/*8* subfamily (Fig 1, S1 Fig) contain largely identical functional domains, share DNA binding specificity, and exhibit functional redundancy [26,27]. For example, mouse gene targeting experiments, in which *PAX2* is replaced by *PAX5* under control of endogenous *PAX2* regulatory elements, show complementation of developmental abnormalities otherwise resulting from *PAX2* deletion [28]. While there is spatiotemporal overlap of *PAX2*/*5*/*8* expression, for instance in parts of the developing nervous system, less overlap occurs in adult tissues [29]. *PAX8 is* expressed predominantly in the adult thyroid and *PAX2* in the adult kidney, where *PAX2* plays a protective role in response to hyperosmolarity encountered by inner medullary cells of nephrons [30]. Only *PAX5* is expressed in lymphocytes.

As neither *PAX2* nor *PAX8* are expressed in lymphocytes, they are unlikely to be subjected to the same selective pressures favoring *PAX5* mutation during leukemogenesis, and, not surprisingly, mutations are not detected in ALL [9]. Therefore, it is not hard to imagine that *PAX2* and *PAX8* could represent intact yet latent functional substitutes for *PAX5* in pre-B ALL. Here we demonstrate the ability of both *PAX2* and *PAX8* to substitute for *PAX5* loss-of-function and reverse the developmental blockade in pre-B ALL cells. We show that restoration of differentiation is similar using all three *PAX* family members and consists of changes to downstream gene expression, cell surface marker expression, cell size, and ultimately cell growth and survival. Additionally, as the translational utility of this strategy is predicated on the ability to activate the endogenous expression of these paralogs in the B cell lineage, we evaluate the aforementioned pathway of response to hyperosmolarity, which plays a prominent role in the kidney. We show that *PAX2* and *PAX5* exhibit transcriptional upregulation in response to hyperosmolarity in pre-B ALL cells, that *PAX2* activation in lymphocytes, as in the kidney, is mediated by the tonicity response enhancer binding protein (*TonEBP/NFAT5)*, and, finally, that hyperosmolarity-driven *PAX2*/*5* activation correlates with changes in B cell developmental gene expression similar to those seen with exogenous *PAX2*/5/8 re-expression.

## Results

### *PAX2* and *PAX8* compensate for *PAX5* loss-of-function by modulating developmental gene expression in pre-B ALL cells

*PAX5* loss-of-function results in B cell developmental blockade and contributes to leukemic transformation [16,17]. As an important early B cell transcription factor, *PAX5* is responsible for both positively and negatively regulating developmental genes, driving differentiation towards a B lymphoid specific fate. Transcriptional targets of *PAX5* are numerous and include B cell receptor (BCR) complex protein *CD79a*, the B cell specific transcriptional regulator *BACH2*, and the canonical B cell specific surface antigen *CD19*.

**Fig 2:**
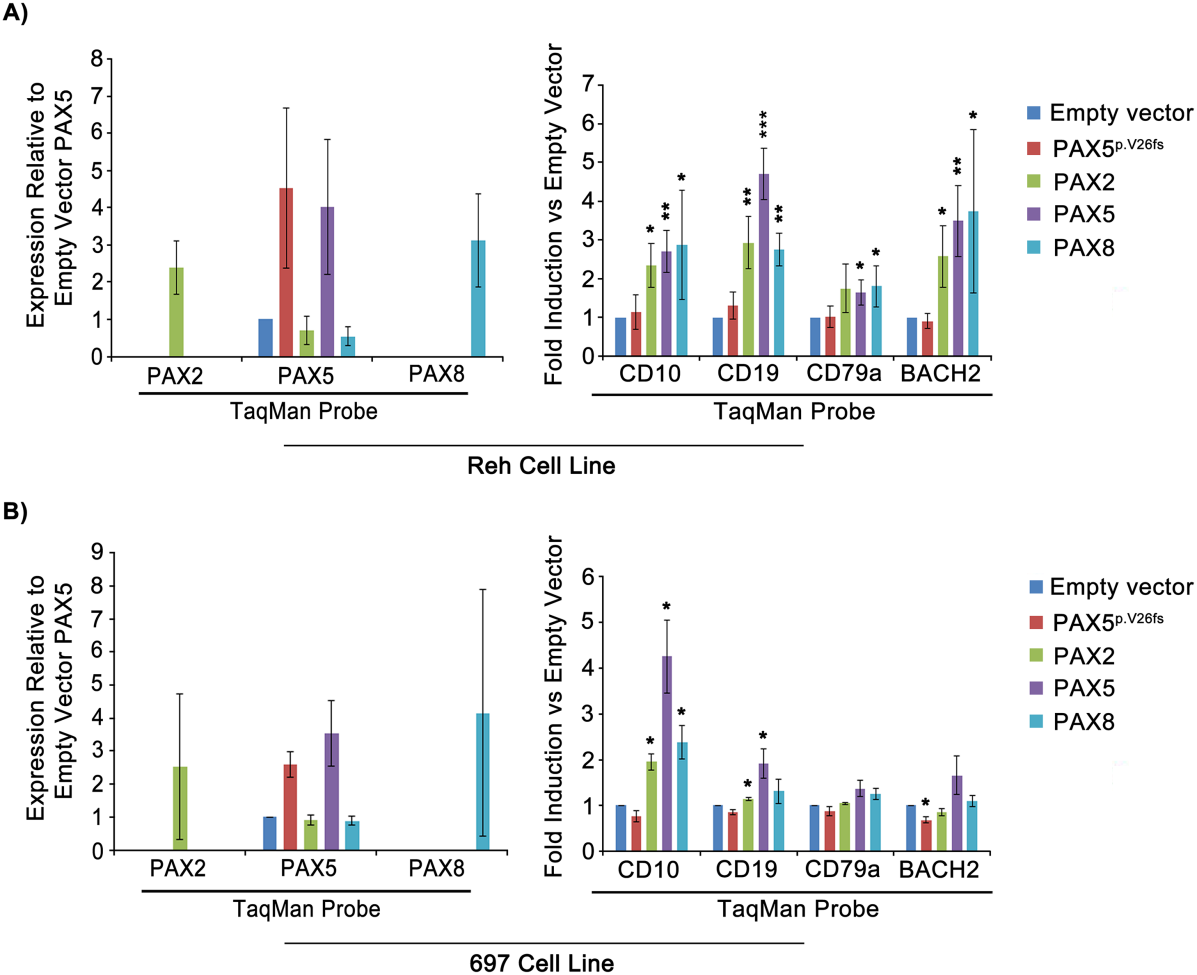
*PAX2* and *PAX8* compensate for *PAX5* loss-of-function by modulating developmental gene expression in pre-B ALL. A) qRT-PCR of RNA/cDNA preparation from FACS of ZsGreen-positive Reh cells transduced with lentivirus containing transgenes indicated in key. B) 697 cells treated/harvested similarly. *PAX2* and *PAX8* levels are presented relative to baseline *PAX5* due to the lack of detectable endogenous *PAX2* or *PAX8* (see Methods). Both A and B are representative of 3 separate experimental replicates. Error bars = standard deviation. Statistical significance derived using one sample t-test vs. empty vector, assuming unequal variation (EV = 1), p-values * <0.05, ** <0.005, *** <0.0005.

We began by confirming recent findings that re-expression of exogenous *PAX5* rescues *PAX5*-deficient pre-B ALL cells [18] and assessing whether exogenous expression of *PAX5* paralogs, *PAX2* or *PAX8*, could function in a similar capacity. We initially evaluated the ability of *PAX5*, *PAX2* or *PAX8* to regulate a subset of *PAX5* transcriptional targets, including *CD79a*, *BACH2*, and *CD19*. We also included *CD10*, which is a marker of B cell differentiation exhibiting a bell-shaped pattern of developmental expression levels that peak at the pro to pre-B cell transition [18,31]. We tested *PAX* factors in Reh cells, which were derived from a primary clonal culture isolated from pre-B ALL peripheral blood [32] and contain a heterozygous p.A322fs *PAX5* null mutation [33]. As a *PAX5* wild type control, we compared 697 cells, which are derived from a primary clonal culture of ALL bone marrow [34] and contain an *E2A(TCF3)*/*PBX1* fusion gene arising from a t(1;19) chromosomal translocation [35]. Cells were stably transduced with lentivirus expressing either full length human *PAX5*, *PAX2*, or *PAX8*, along with a fluorescent marker, ZsGreen, driven from an internal ribosomal entry site (IRES). As a functionally negative control, we used a vector expressing the clinically observed pre-B ALL *PAX5* null mutation, *PAX5*^p.V26fs^ [36]. At day 4 following transduction, 2×10^5^ ZsGreen-positive cells of each transduction type were sorted by FACS (see S2 Fig for gating strategy). Using quantitative real time PCR, we found that transgene expression of *PAX5*, *PAX2*, or *PAX8* in both Reh and 697 cells led to significant upregulation of *PAX5* target gene expression, relative to empty vector or the negative control *PAX5*^p.V26fs^. With the exception of *CD10*, which is not a known *PAX5* transcriptional target, this upregulation was more pronounced in *PAX5*-mutant Reh cells compared to *PAX5*-wild type 697 cells (Fig 2A and 2B, respectively).

### *PAX2* and *PAX8* rescue immunophenotypic advancement of B cell differentiation in pre-B ALL cells

**Fig 3:**
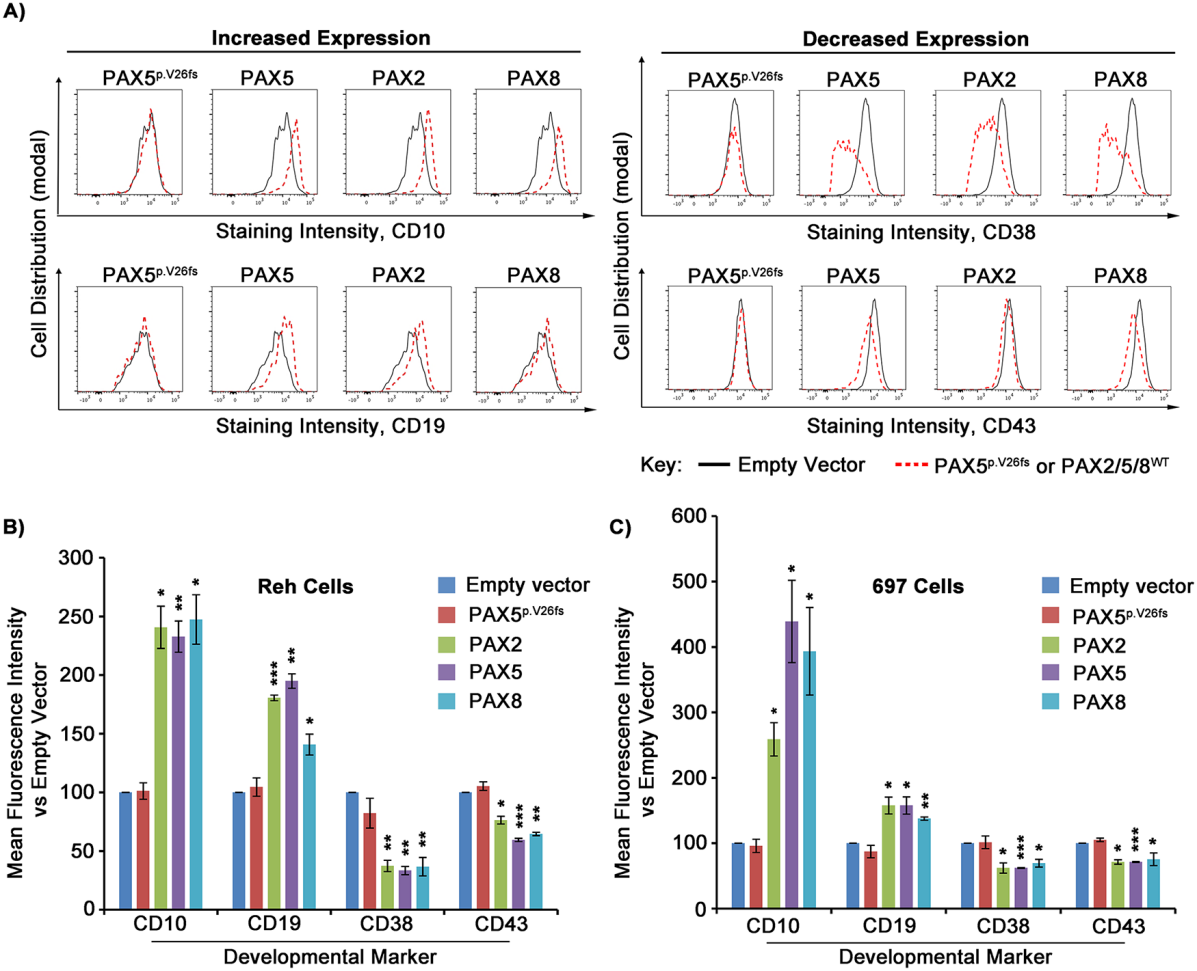
*PAX2* and *PAX8* rescue immunophenotypic advancement of B cell differentiation in *PAX5* loss-of-function pre-B ALL cells. A) Representative histogram comparisons of developmental marker antibody staining intensity for ZsGreen-positive Reh cells transduced with lentivirus containing either empty vector (black outlines) or indicated *PAX* mutant or wild type transgenes (red-dotted outlines). Antibodies used for flow cytometry denoted beneath each panel. B) Relative mean fluorescence intensity for each antibody, from A, average of 3 separate experimental replicates each for Reh cells, and C) for 697 cells. Values relative to empty vector transduced cells. Error bars = standard deviation. Statistical significance derived using one sample t-test vs. empty vector (EV = 100%), p-values * <0.05, ** <0.005, *** <0.0005.

To further evaluate the ability of *PAX2* and *PAX8* to rescue *PAX5* loss-of-function in pre-B ALL cells, we assessed whether their transcriptional redundancy resulted in enhanced immunophenotypic progression by comparing their ability to modulate a subset of surface markers of B cell differentiation. CD10 (CALLA) and CD19 are surface markers found on normal, as well as leukemic, pre-B cells. Increases in both markers are expect to accompany B cell differentiation, whereas CD38 and CD43 are both downregulated during the large-to-small pre-B cell transition [18,37]. Reh and 697 pre-B ALL cells were transduced with lentivirus expressing *PAX*-IRES-ZsGreen, as before. At day 4 post-transduction, cells were stained with antibodies for cell surface markers, followed by analysis of ZsGreen-positive cells using flow cytometry. Cells expressing *PAX5*, *PAX2*, or *PAX8* constructs showed significantly upregulated levels of CD10 and CD19 with downregulated levels of CD38 and CD43, relative to cells transduced with either empty vector or *PAX5*^p.V26fs^ (Fig 3A and 3B). These results demonstrate a level of functional phenotypic rescue beyond simple transcriptional activation and show a shared ability within the *PAX2*/5/8 subfamily to promote immunophenotypic changes associated with advanced differentiation in pre-B ALL. Interestingly, *PAX5*-wild type 697 cells again exhibited similar results (Fig 3C, note scale of intensity).

### Exogenous *PAX2* and *PAX8* reduce replicative potential and promote physical changes characteristic of the large-to-small B cell transition

The large-to-small pre-B cell transition occurs just prior to the emergence of the immature B cell and marks the end of the heavily proliferative large pre-B cell state, resulting instead in a population of pre-B cells which are not only smaller, as the name suggests, but also less proliferative [38]. As noted, we observed that transduction of Reh and 697 cells with *PAX* paralogs led to decreases in expression of CD38 and CD43, which are both downregulated during this transition [18,37]. This observation suggested that, consistent with prior observations related to *PAX5* re-expression in Reh cells [18], *PAX2* and *PAX8* could advance differentiation in these cells, driving them through the large-to-small transition and ultimately to a normal, more quiescent state. To address this possibility, we analyzed changes in cell size as well as effects on replicative potential following transduction with *PAX* factors.

**Fig 4:**
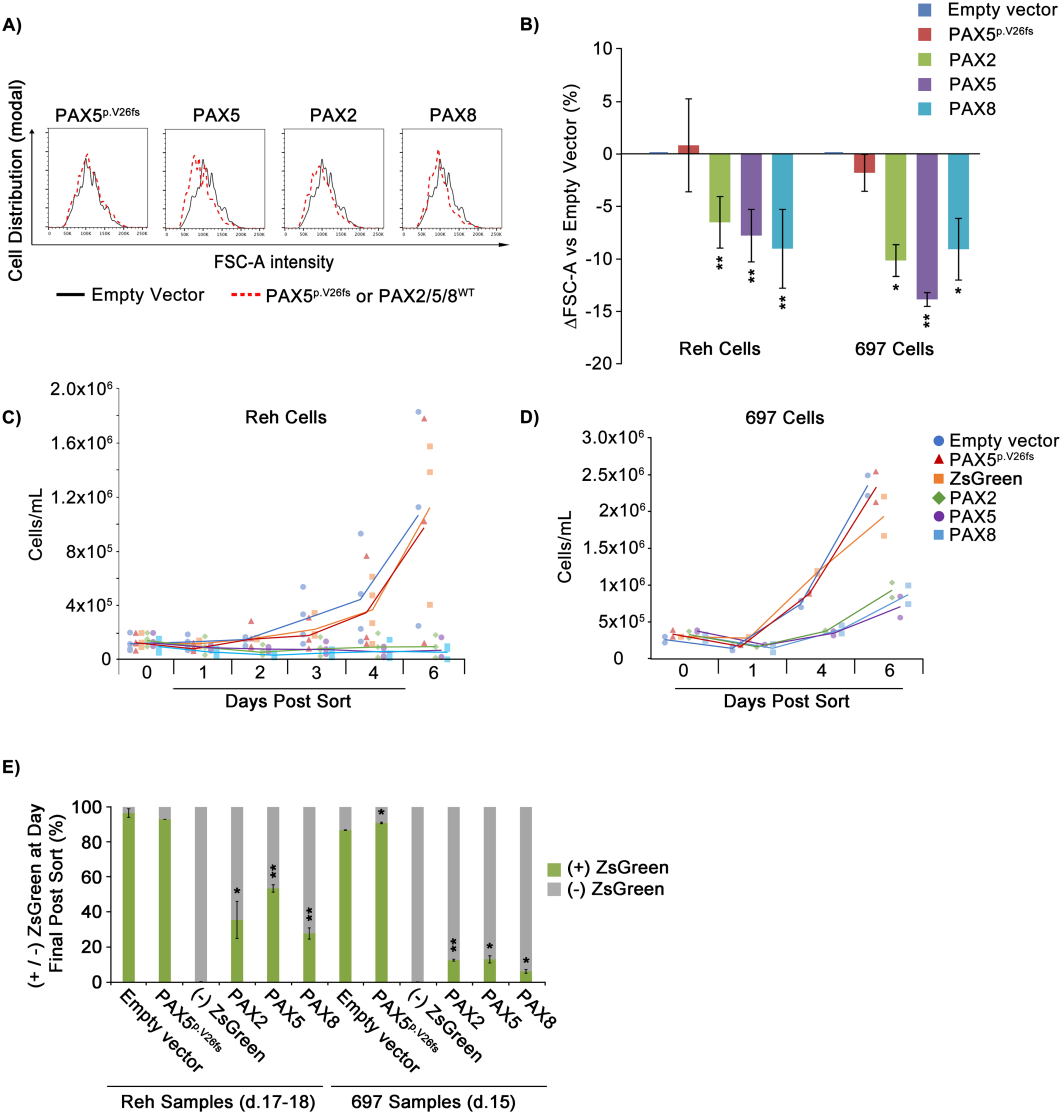
*PAX2* and *PAX8* promote developmentally characteristic large-to-small B cell transition and exit from the cell cycle in *PAX5*-deficient pre-B ALL cells. A) Representative histogram of FSC-A intensity for empty vector (black outlines) vs. *PAX* transduced (red-dotted outlines) Reh cells. B) Percent deviation from empty vector (set to 0) of mean FSC-A values for cells expressing indicated *PAX* mutant or wild type transgenes (see key) for an averaged 6 and 3 experimental replicates for Reh and 697 cells, respectively. C) Reh (3 experimental replicates) and D) 697 (2 experimental replicates) cell culture density vs. time, following sorting (day 4 post transduction) for ZsGreen-positive cells expressing indicated transgenes. Data points for all replicates are shown, along with lines fitting the mean values for each treatment. (-) ZsGreen cells represent unsuccessfully transduced cells sorted from the *PAX5* lentivirus exposed cell suspension. E) Percentage of ZsGreen positive vs. negative cells at 11–14 days post sort for ZsGreen for 2 experimental replicates for per cell line. Error bars = standard deviation. Statistical significance derived using one sample t-test vs. empty vector, p-values * < .05, ** < .005, *** < .0005. See also S3, S4 and S5 Figs.

The flow cytometry parameter of forward scatter area (FSC-A) is a widely accepted proxy for estimating cell size [39]. Similar to *PAX5*, exogenous expression of *PAX2* and *PAX8* led to a reduction in Reh cell FSC-A ranging from 7–10.1%, relative to either empty vector or *PAX5*^p.V26fs^ negative control (Fig 4A and 4B). Again, 697 cells displayed similar results (Fig 4B). However, as a negative control, the human embryonic kidney cell line, HEK293T, transduced with *PAX2*/*5*/*8* or controls, did not exhibit a shift in cell size (S3C Fig).

We next evaluated the effect of exogenous *PAX* paralog expression on the long-term replicative potential of Reh and 697 cells. Cells were transduced with *PAX5*, *PAX2*, *PAX8*, empty vector, or *PAX5*^p.V26fs^ negative control. At day 4 post-transduction, 2×10^5^ cells of each group were FACS-sorted for ZsGreen (at ~98% purity, see S2 Fig for gating strategy) and returned to culture. For the following 6 days, daily measurement of culture density, performed in duplicate using a hemocytometer, allowed us to compile growth curves for all groups. While control cultures expanded normally, *PAX* paralog expression resulted in a complete inhibition of culture expansion in Reh cells (Fig 4C, S4A Fig). Growth inhibition was also present, but less complete, in 697 cells (Fig 4D, S4B Fig) and largely absent in HEK293T control cells (S3B Fig). From this point, it became necessary to periodically passage cultures in order to maintain viable cell densities (i.e., 2×10^5^–2×10^6^ cells/mL). At days 11–14, we again used flow cytometry to measure ZsGreen-expressing cell populations. Cultures transduced with *PAX* paralogs exhibited dramatically reduced ZsGreen expression as a percentage of total cells, ranging from 28–54% in Reh cells and 6–13% in 697 cells, whereas both the empty vector and *PAX5*^p.V26fs^ control groups maintained expression in ~90% of cells (Fig 4E and S4C Fig). Growth inhibition and the reduced proportion of ZsGreen-positive cells together suggest that these cells reduce their rate of growth and are outgrown by the ~2% of ZsGreen negative cells initially harvested by mis-sorting and/or that *PAX*/ZsGreen-positive cells die out so that only ZsGreen-negative cells remain and continue to grow. In support of the latter interpretation, *PAX* gene expression led to an apparent delay in cell cycle progression and conferred a modest increase in apoptosis, as measured by flow cytometry analysis of DNA content (with DAPI staining) and Annexin V staining, respectively (S5 Fig).

We have therefore confirmed previously published literature showing that restoration of *PAX5* levels rescues deficiency of *PAX5* activity in pre-B ALL cells [18] and have shown for the first time that its paralogs, *PAX2* and *PAX8*, demonstrate a high level of functional redundancy in downstream activation of B cell specific gene expression, promoting differentiation similar to that seen with *PAX5*.

### Extracellular hyperosmolarity induces endogenous *PAX2* and upregulates *PAX5* in Reh cells

The observation that *PAX2* and *PAX8* can rescue the *PAX5* loss-of-function differentiation blockade in pre-B ALL cells suggests their activation in vivo could represent a potential therapeutic strategy. In such a context, the use of small molecules to induce their endogenous expression would be useful.

**Fig 5:**
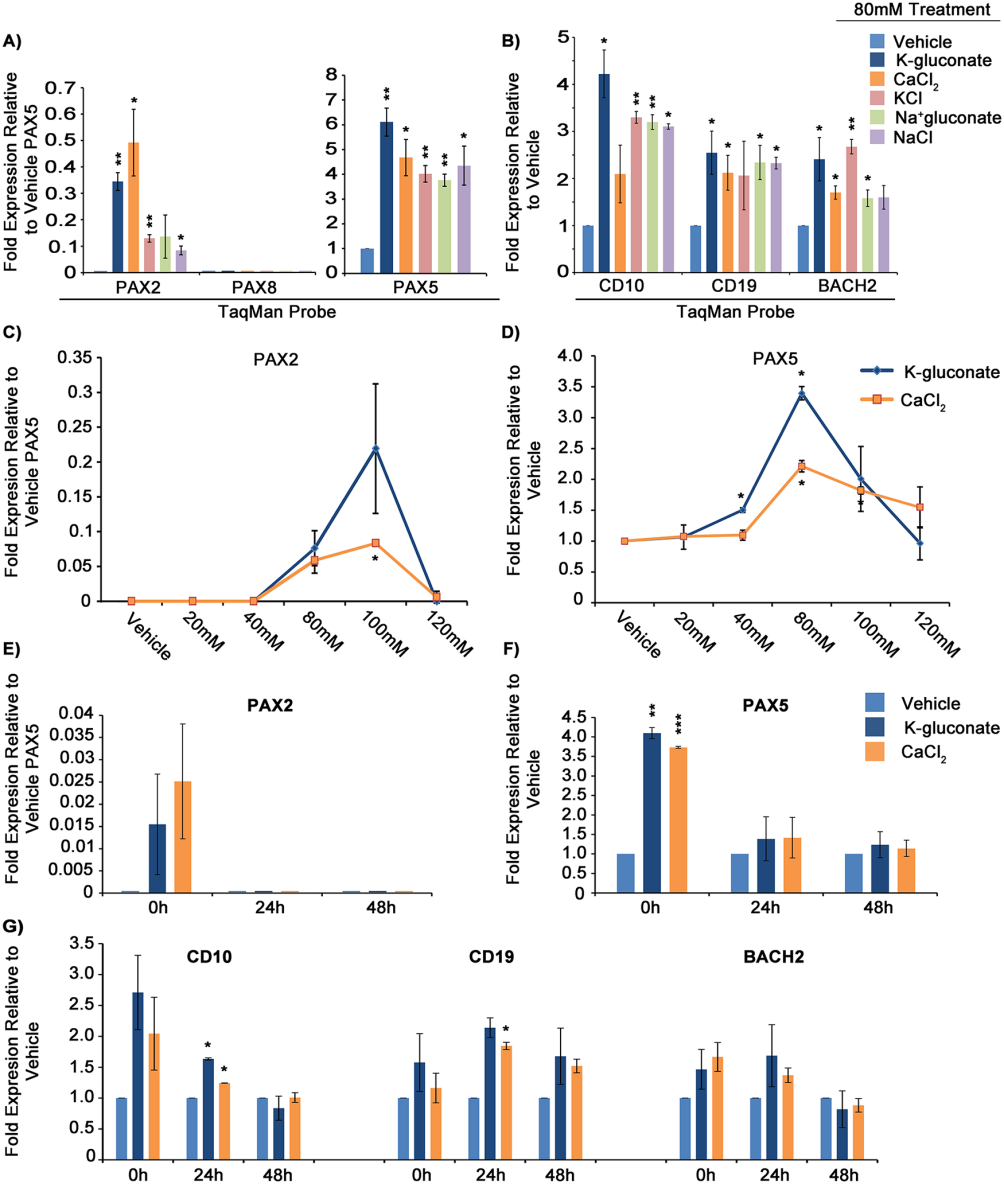
Extracellular hyperosmolarity induces endogenous *PAX2* and *PAX5* expression in pre-B ALL cells. A) Fold-expression of *PAX2*, *PAX8* (none detected), and *PAX5* mRNA in response to 24 hour exposure to 80mM treatments of indicated compounds in Reh cells. B) Fold-expression of downstream markers in response to treatments in A. C) Dose-response curve in Reh cells showing *PAX2* and D) *PAX5* mRNA expression in response to varying K-gluconate and CaCl_2_ concentrations. E) Relative *PAX2*, F) relative *PAX5*, and G) relative downstream gene levels following pulse chase, where x-axis represents the incubation time in normal media following 24 hours incubation in 80mM K-gluconate or CaCl_2_ and flow sorting for live cells via FSC-A/SSC-A. *PAX2* values shown are 2^-ΔΔC_T_^, relative to vehicle-treated *PAX5* levels (as there is no detectable baseline *PAX2* expression). *All other* gene expression values are 2^-ΔΔC_T_^, relative to corresponding vehicle expression values. Error bars = standard deviation. Statistical significance derived using one sample t-test vs. vehicle treated, assuming unequal variation (vehicle = 1), p-values * <0.05, ** <0.005, *** <0.0005. A and B are each 3 averaged experimental replicates while C-G are each 2. All values shown are relative to *ACTB* as endogenous reference gene. See also S6A–S6C Fig for *PAX* amplification curves, S7A and S7B Fig for *GAPDH* normalized dose-response curves, and S7C and S7D Fig for 697 dose response to K-gluconate.

In attempting to identify drugs capable of activating endogenous *PAX2* or *PAX8* we initially surveyed a variety of agents targeting epigenetic repressive marks or that have been reported to promote lymphocyte differentiation; however, none induced detectable *PAX2* or *PAX8* expression. We then evaluated compounds known to induce *PAX* family gene expression in other model systems. Manipulation of transmembrane voltage potential in *Xenopus laevis* activates transcription factors, including *PAX6*, resulting in ectopic eye formation [40]. Based on this observation, we tested a variety of hyper- and hypo-polarizing compounds for their ability to induce *PAX2* and/or *PAX8* expression in Reh cells. We found that 24 hour exposure to membrane depolarizing concentrations (80mM) of C_6_H_11_KO_7_ (K-gluconate) in cell media led to induction of *PAX2* expression to as much as 0.3 fold of baseline *PAX5*, as measured by qRT-PCR. Interestingly, significant upregulation of *PAX5* expression was also observed (Fig 5A and S6A, S6B and S6C Fig).

While such concentrations of K-gluconate are known to induce membrane depolarization [41], treatment with monensin and other compounds that are also known to promote membrane hypopolarization did not influence expression of *PAX* genes. As both potassium and gluconate ions are potentially capable of independent interaction with membrane channels or other cellular machinery that could influence downstream gene expression [42], we tested a variety of salts containing these and other ions, for their ability to influence *PAX* expression. Surprisingly, 80mM concentrations of NaC_6_H_11_O_7_ (Na-gluconate), KCl, CaCl_2_, and NaCl all promoted detectable induction of both *PAX2* at 0.08–0.5 fold and *PAX5* at 3.8–6.1 fold, relative to baseline *PAX5* (Fig 5A). Evaluation of downstream *PAX5* target and developmental marker genes, *CD19*, *BACH2*, and *CD10*, demonstrated concurrent upregulation at levels similar to those seen with transgene-driven exogenous *PAX* expression (Fig 5B). While the ionic composition of these agents differs, a commonality is that they all increase the osmolarity of cell growth media.

We observed quantitative differences in the ability of these osmolytes to induce *PAX2*/*5*, perhaps due to their variable ability to penetrate the cell membrane, utilizing channels specific for their uptake or efflux. As such, based on their greater relative ability to upregulate both *PAX2* and *PAX5* in Reh cells, we selected K-gluconate and CaCl_2_ for further evaluation. Dose-response curves revealed that 80-100mM concentrations (corresponding to ~400-540mOsmol/kg H_2_O in RPMI media) were optimal for either salts’ ability to upregulate *PAX2* and *PAX5*, with little activity occurring at lower concentrations (Fig 5C and 5D, and S7A and S7B Fig). Similar results were seen with 697 cells; however, the magnitude of induction was less than that observed in Reh cells (S7C and S7D Fig).

In studying the kinetics of this response to hyperosmolarity, 24 hour exposure to high salt concentrations, followed by sorting of live cells and return to normal media for extended incubation revealed that both *PAX2* and *PAX5* upregulation occurred quickly, but decreased within 24 hours post exposure to salt (Fig 5E and 5F). While *CD10* followed a similar temporal pattern to *PAX* gene modulation, increases in direct *PAX5* target genes *CD19* and *BACH2* were delayed and more persistent, supportive of their sequential response to *PAX* induction following hyperosmolarity, rather than to hyperosmolarity alone (Fig 5G). Interestingly, the RNA collection method affected the magnitude of induction for *PAX2*, which was as much as 10-fold greater in RNA extracted from cells immediately following treatment compared to RNA harvested from cells which were first treated, then sorted for viability (as assessed by FSC-A/SSC-A). In contrast, induction of *PAX5* appeared to be similar regardless of the RNA collection method. (RNA collection methods are described in Figure Legends and Methods.) This observation suggests an interplay between cell viability and *PAX2* expression (Fig 5A and 5C, compared to Fig 5E; see also S2 Fig for gating strategy).

### Global gene expression reinforces *PAX2/5/8* functional similarity with regard to B cell development and highlights overlapping effects from the response to hyperosmolarity

Using cell surface markers, morphological changes, and a subset of *PAX5* transcriptional targets, we have demonstrated the ability of *PAX2* and *PAX8* to rescue *PAX5* loss-of-function in pre-B ALL cell lines. To evaluate the full extent to which *PAX2* and *PAX8* can substitute for *PAX5*, as well as to compare *PAX* transgene expression with the response to hyperosmolarity, we evaluated global changes in gene expression by RNA sequencing (RNA-seq) following *PAX2*, *PAX5*, or *PAX8* transfection or treatment with 80mM K-gluconate or CaCl_2_ in Reh cells.

**Fig 6:**
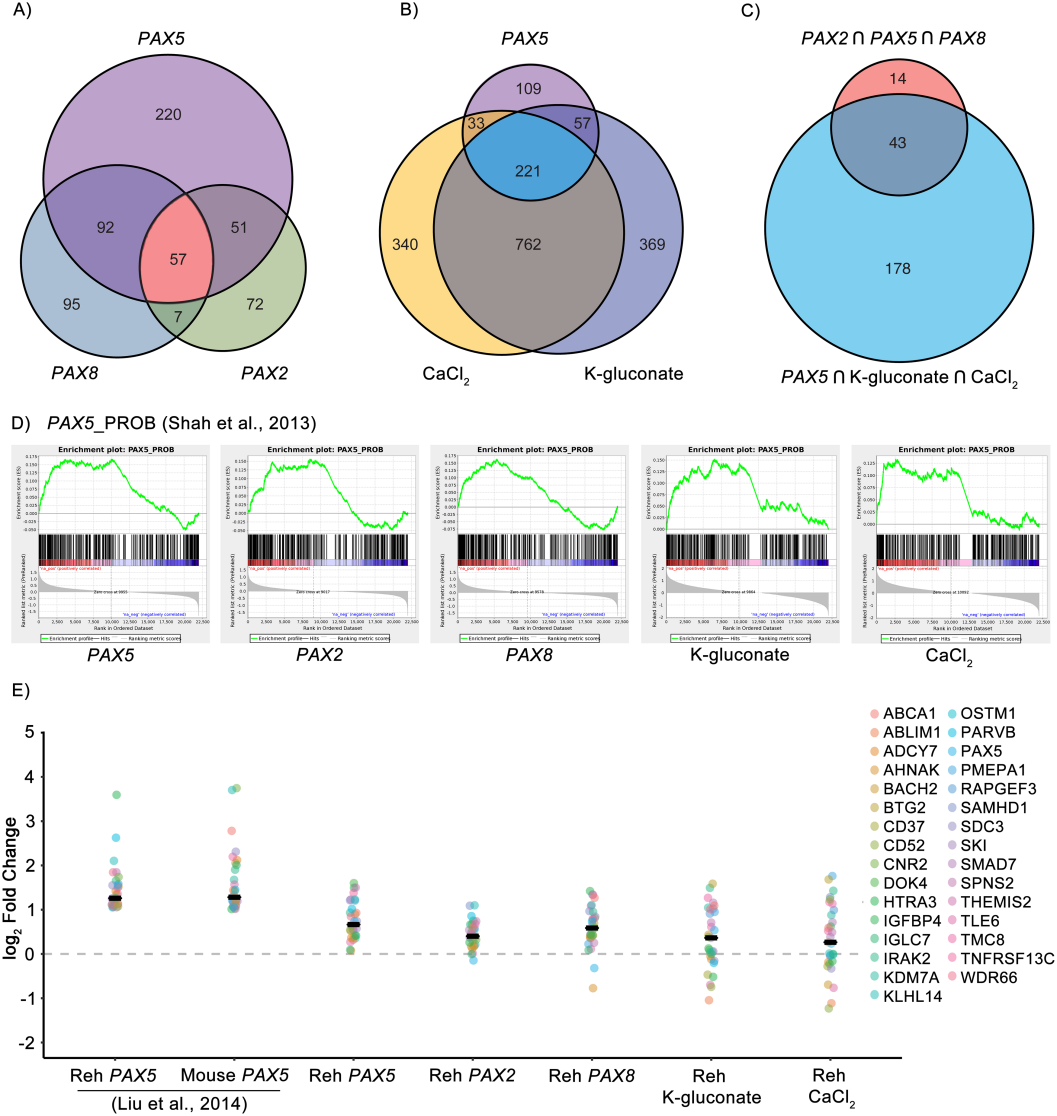
RNA-seq data shows that *PAX2*, *PAX8*, K-gluconate, and CaCl_2_ affect pathways modulated by the restoration of *PAX5*. A) Venn diagram of enriched gene sets in Reh samples transfected with *PAX5*, *PAX2*, or *PAX8*. B) Venn diagram of enriched gene sets in Reh cells transfected with *PAX5* or exposed to either CaCl_2_ or K-gluconate (80mM). C) Venn diagram of common *PAX2∩PAX5∩PAX8* and common *PAX5∩*CaCl_2_*∩*K-gluconate enriched gene sets. D) Gene set enrichment plots for the *PAX5*_PROB gene set adapted from [13]. E) Changes in expression of genes regulated by PAX5 in Reh cells and that are related to remission of B-ALL in mice. A black bar indicates the median gene log_2_ fold change for each sample. Liu et al. samples were first reported in [18]. See also S8–S11 Figs.

Gene set enrichment analysis (GSEA) revealed common enrichment pathways based on biological process and transcription factor targets (Fig 6). Gene sets previously shown to be either direct transcriptional targets of PAX5 at the pro and mature stages of B cell development or whose regulation relies on PAX5 mediation of differentiation from the pro to mature B cell stages displayed enrichment as well [13,43]. We restricted analysis to gene sets with a false discovery rate less than 0.05.

**Fig 7:**
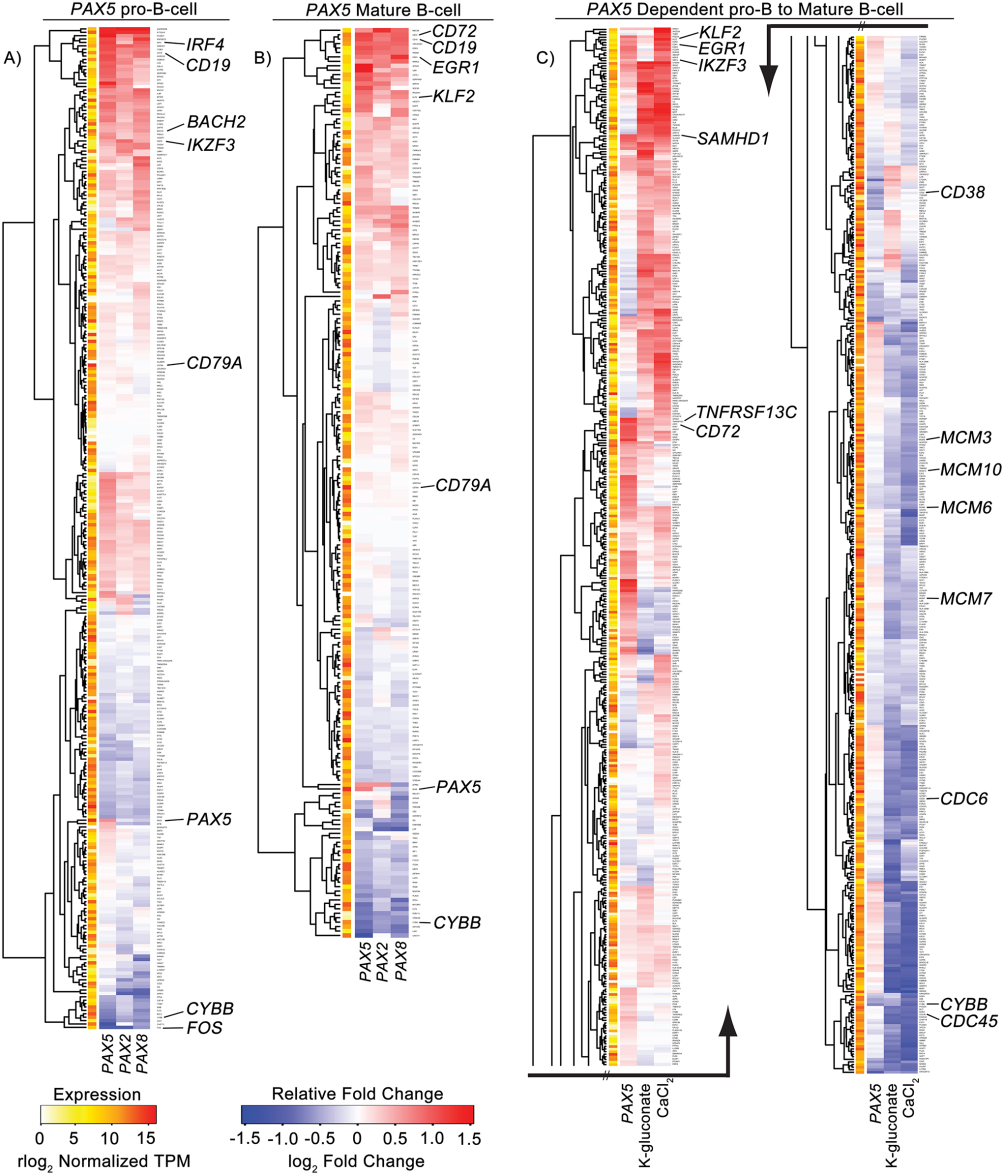
Increasing *PAX* expression or osmolarity changes expression of genes related to B cell development. A) Fold change heatmap of *PAX5* related pro-B cell genes with *PAX5* binding sites in the promoter. B) Fold change heatmap of *PAX5* related mature B cell genes with *PAX5* binding sites in the promoter. C) Fold change heatmap of genes involved in the *PAX5* dependent transition of pro-B cells to mature B cells. Average gene expression across samples is illustrated to the left of each heatmap. The pro-to-mature B cell heatmap has been cut in half and displayed side-by-side due limited space. See also S8–S11 Figs.

We observed enrichment of 420 gene sets in Reh cells transfected with *PAX5*. 35% (149) or 26% (108) of these gene sets are also enriched in *PAX2* or *PAX8* transfected cells, respectively, with 14% (57) common to all three samples (Fig 6A). The majority of these gene sets involve genome accessibility and protein translation (e.g., methylation, peptidyl lysine modification, translational initiation, and cytoplasmic translation), but we also see negative enrichment of known cell cycle regulation transcription factor gene sets such as those involving MYC/MAX and E2F1 (MYCMAX_01 and E2F1_Q4, respectively, S1 Table). *PAX2*/*5*/*8* transfected samples also show similar enrichment patterns in the PAX5 B cell developmental gene sets (Fig 7A and 7B, S2 Table and S1 Dataset), each factor promoting the upregulation of *CD72*, *IRF4*, *BACH2*, *CD19*, *EGR1*, *IKZF3*, *KLF2*, and *SAMHD1* as well as the suppression of *CYBB* and *FOS*.

Interestingly, K-gluconate and CaCl_2_ share a larger percentage of the *PAX5* enriched gene sets, 66% (278) and 60% (254), respectively, than either *PAX2* or *PAX8* transfected samples (Fig 6B). 53% (221) of the *PAX5* enriched gene sets are also enriched following both CaCl_2_ and K-gluconate exposure (S1 Table). In general, there is a larger, overlapping response when comparing the two salt treatments, presumably part of a general response to hyperosmolarity. Of note, gene sets related to the transport of calcium ions, chloride ions, potassium ions, and organic anions, as well as cytosolic calcium regulation, are positively enriched for all three treatments—an expected result for cells exposed to K-gluconate and CaCl_2_, but not for forced expression of *PAX5*. Again, the MYCMAX_01 and E2F1_Q4 gene sets are negatively enriched, linking increasing osmolarity with a pathway for reduced proliferation and a decrease in B cell size [44], although leading edge analysis of the gene sets suggests different genes responsible for enrichment when compared to *PAX2*/*5*/*8* (S3 Table). Both CaCl_2_ and K-gluconate show their strongest *PAX5* related response in the pro to mature B cell transition gene set (Fig 7C and S2 Table). Here overlapping clusters of upregulated genes similar to the individual pro and mature B cell gene sets (e.g., *KLF2*, *EGR1*, *IKZF3*, *SAMHD1*, and *CD72*) are highlighted. *TNFRSF13C/BAFF-R*, a regulator of peripheral B cell survival, is also upregulated, whereas downregulated genes include cell cycle initiation factors *CDC6* and *CDC45* and pre-replication complex components *MCM3*, *MCM6*, *MCM7*, and *MCM10*.

In total, 43 of the 57 gene sets common to *PAX2*/*5*/*8* transfected samples are also enriched in the CaCl_2_ and K-gluconate treated samples, corresponding to 10% of the total enriched gene sets in *PAX5* transfected Reh cells (Fig 6C, S1 Table). Most of the enriched sets common for both *PAX2*/*5*/*8* transfectants and salt treatment again relate to genome structure and protein synthesis and also similarly include MYCMAX_01 and E2F1_Q4 transcription factor targets. Both the pro-B cell and pro to mature B cell gene sets are enriched in all samples, as well. The greatest similarity across treatment conditions is seen in the pro-B cell set of genes (Fig 6D), with the only difference being a lack of negative enrichment of genes in either CaCl_2_ or K-gluconate treated cells. Overall, these results suggest that B cell maturation is regulated by a set of genes and pathways commonly responsive to either *PAX* gene expression or hyperosmolarity.

Liu *et al*. [13] restored *PAX5* expression in Reh cells and compared global changes in gene expression via RNA-seq to gene expression in a *Pax5*-deficient/*Stat5*-activated mouse model of ALL. They identified 31 genes in Reh cells, upregulated by greater than two-fold in response to exogenous *PAX5*, that are also commonly upregulated with restoration of *Pax5* in the mouse model of ALL. Restoration of *Pax5* in this model triggers durable disease remission. The log_2_ fold change values we observed for these 31 genes in *PAX2*/*5*/*8* transfected and CaCl_2_ or K-gluconate treated cells appear in Fig 6E, charted alongside corresponding original data from Liu *et al*. Although treatment windows for our samples were somewhat brief compared to duration of *Pax5* induction in mice, we found similar increases in relative expression across this set of 31 genes, albeit at levels roughly half of what Liu *et al*. reported. These data demonstrate that *PAX* paralog expression or hyperosmolar treatment both similarly modulate an important subset of genes associated with disease remission when *PAX5* expression is restored to normal levels in cell and mouse models of *PAX5*-deficient ALL.

To confirm RNA-seq results, we used qRT-PCR to validate the responses of several genes where the heatmap clustering showed them to be upregulated by at least 4 of 5 treatment conditions, along with an additional gene, *SNX12*, which was slightly downregulated by 4 of 5 conditions. qRT-PCR analysis of all 7 of these genes accurately corroborated the trends seen in the RNA-seq data (S8A Fig). Notably, relative to RNA-seq, magnitudes of induction (if present) were almost always greater using qRT-PCR ΔΔCT values. This is likely due to the conservative estimates of differential expression from the DESeq2 normalization algorithm we employed to analyze RNA-seq data. Nevertheless, trends were consistent regardless of technique or genes referenced for comparison.

### TonEBP/NFAT5 modulates *PAX2* but not *PAX5* upregulation in response to hyperosmolarity, revealing the NFAT5 pathway as a target for activating endogenous *PAX2* expression in pre-B ALL

**Fig 8:**
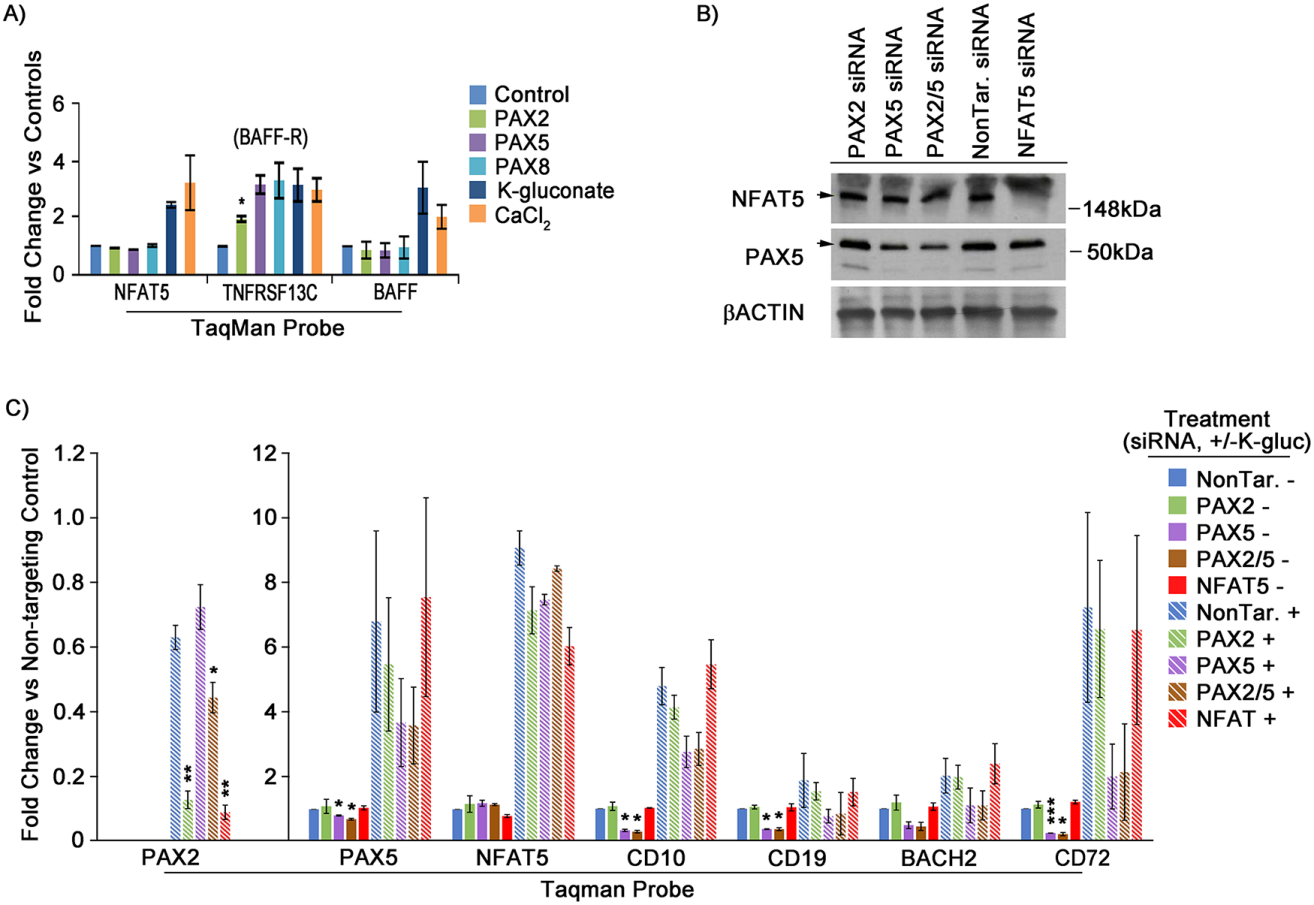
NFAT5 plays a role in hypertonicity mediated *PAX2* expression. A) qRT-PCR validation of RNA-seq data for *NFAT5*, *BAFF-R*, and *BAFF*. Graphs represent the average of two separate experimental replicates. Fold change values are 2^-ΔΔC_T_^, relative to each samples’ respective control (i.e., empty vector or untreated), with *ACTB* used as endogenous reference gene. B) Representative western blot of PAX5 and NFAT5 protein knockdown by siRNA. C) Fold expression of *PAX2* (left scale), *PAX5*, *NFAT5*, and downstream genes (right scale) after treatment with (+/-) 80mM K-gluconate and (+/-) siRNA knockdown of *PAX2*, *PAX5*, *PAX2/5*, or *NFAT5*, for 3 experimental replicates. *PAX2* values are 2^-ΔΔC_T_^, relative to vehicle-treated *PAX5* levels. All other gene expression values are 2^-ΔΔC_T_^, relative to corresponding vehicle expression values. Protein lysates in part B were bulk harvested from treated cells while RNA in part C was isolated from live cells first flow sorted by FSC-A/SSC-A. Error bars = standard deviation. Statistical significance derived using one sample t-test vs. control treated, assuming unequal variance (i.e., vehicle = 1), p-values * <0.05, ** <0.005, *** <0.0005. See also S9 Fig for TonE elements at *PAX2* and *PAX5* loci, as well as S8 Fig for NFAT5 target solute channels in response to *NFAT5* siRNA knockdown.

Cellular response to hypertonicity, as brought about by hyperosmolarity, is thought to be largely mediated by the tonicity-responsive enhancer binding protein, *TonEBP* [45]. *TonEBP*, also called (and referred to here as) *NFAT5* (nuclear factor of activated T cells 5), is a transcription factor predominantly associated with the kidney but which is also expressed in other tissues, including B cells and, as its name suggests, T cells. Initial response to hypertonicity by NFAT5 involves post-translational modification via phosphorylation, followed by transcriptional activity, including self-induction. Interestingly, NFAT5 mediated gene regulation in the high salt environment of nephrons has been shown to include elevated *PAX2* expression, seemingly as part of a survival mechanism during osmotic stress [30]. Not surprisingly, our RNA-seq data showed that hyperosmolarity in Reh cells led to induction of *NFAT5*, as well as several of its downstream targets (S1 Dataset), consistent with the notion that hyperosmolar concentrations of K-gluconate and CaCl_2_ generate a canonical response to hypertonicity (i.e., an increase in osmotic pressure gradient across the cell membrane). Subsequent evaluation by qRT-PCR confirmed that *NFAT5* mRNA levels, as well as a downstream target associated with B cell maturation, B cell activating factor (*BAFF*), along with its receptor, *TNFRSF13C* (*BAFF-R*) [46], were upregulated in Reh cells after 24 hour treatment with 80mM K-gluconate or CaCl_2_ (Fig 8A). *BAFF-R* alone was also upregulated by *PAX* transgene expression.

Analysis of the 5’ enhancer/promoter regions of both *PAX2* and *PAX5*, along with their intronic and exonic DNA, indicated numerous iterations of the consensus (TGGAAANNYNY) TonE binding site (S9A and S9B Fig) [47]. To determine whether *NFAT5* was involved in hyperosmolarity-induced expression of *PAX2* and *PAX5* and to concurrently assess whether such *PAX* upregulation directly affected downstream gene modulation, we performed siRNA knockdown of these three genes (Fig 8B and 8C). We found that siRNA knockdown of *NFAT5* was sufficient to abrogate *PAX2* upregulation in response to 80mM K-gluconate in Reh cells (Fig 8C). Similarly, knockdown of *NFAT5* quenched hyperosmolarity mediated increases in the solute carriers, *SLC5A3* and *SLC6A6*, both of which are known targets of *NFAT5* (S8B Fig) [48]. Interestingly, neither *PAX5* nor *PAX5* downstream genes upregulated in response to hyperosmolarity were affected by *NFAT5* knockdown (Fig 8C), consistent with a separate, NFAT5 independent mechanism for induction of *PAX5* or, at least, reduced sensitivity of *PAX5* to changes in NFAT5 levels. Importantly, knockdown of *PAX5* itself led to reductions in expression of the downstream genes we assessed, while siRNA directed against *PAX2* had little effect (Fig 8C), suggesting that hypertonic induction of residual wild type *PAX5* expression outweighs *PAX2* with respect to regulation of their common targets. We note that *PAX2* expression is detectable as a transcript, but insufficient to measure at the protein level by western blot.

### Hyperosmolarity stimulates expression of both wild type and mutant *PAX5*

The *PAX5* mutation in Reh cells creates a frameshift leading to premature termination and is thus expected to be subject to nonsense-mediated decay. However, western blot indicates that, in addition to a full-length PAX5 protein corresponding to the wild type allele, a truncated polypeptide that is likely non-functional is apparently generated from the mutant allele, albeit at reduced abundance, suggesting that nonsense-mediated decay is incomplete (as evident in Fig 8B, where both products are specifically targeted by siRNA directed against *PAX5*). Reh cells should therefore contain mRNA from both the wild type and mutant *PAX5* alleles. To determine if either salt treatment differentially activates the wild type as opposed to the mutant *PAX5* allele in Reh cells, we analyzed RNA-seq data and compared the total number of reads obtained from each allele (and that also include the distinguishing mutation). In untreated cells, 27 of 105 total, non-normalized reads (26%) corresponded to transcripts from the mutant allele. In K-gluconate or CaCl_2_ treated cells, the equivalent proportions of mutant transcripts were 54/261 (21%) and 36/135 (27%), respectively. (Using a two-tailed test to compare two population proportions, for untreated versus K-gluconate treated cells, the Z-score is 1.05 and p-value is 0.29. The same comparison for CaCl_2_ treated cells yields a Z-score of -0.17 and p-value of 0.87). These differences are not significant. We conclude that neither salt treatment discriminates between wild type and mutant allele when activating *PAX5* expression, as reflected in proportionately increased total read counts.

### Primary cell response to K-gluconate and efficacy of near clinically achievable mannitol dosage in Reh cells supports the therapeutic potential of targeting hypertonicity response pathways in pre-B ALL

**Fig 9:**
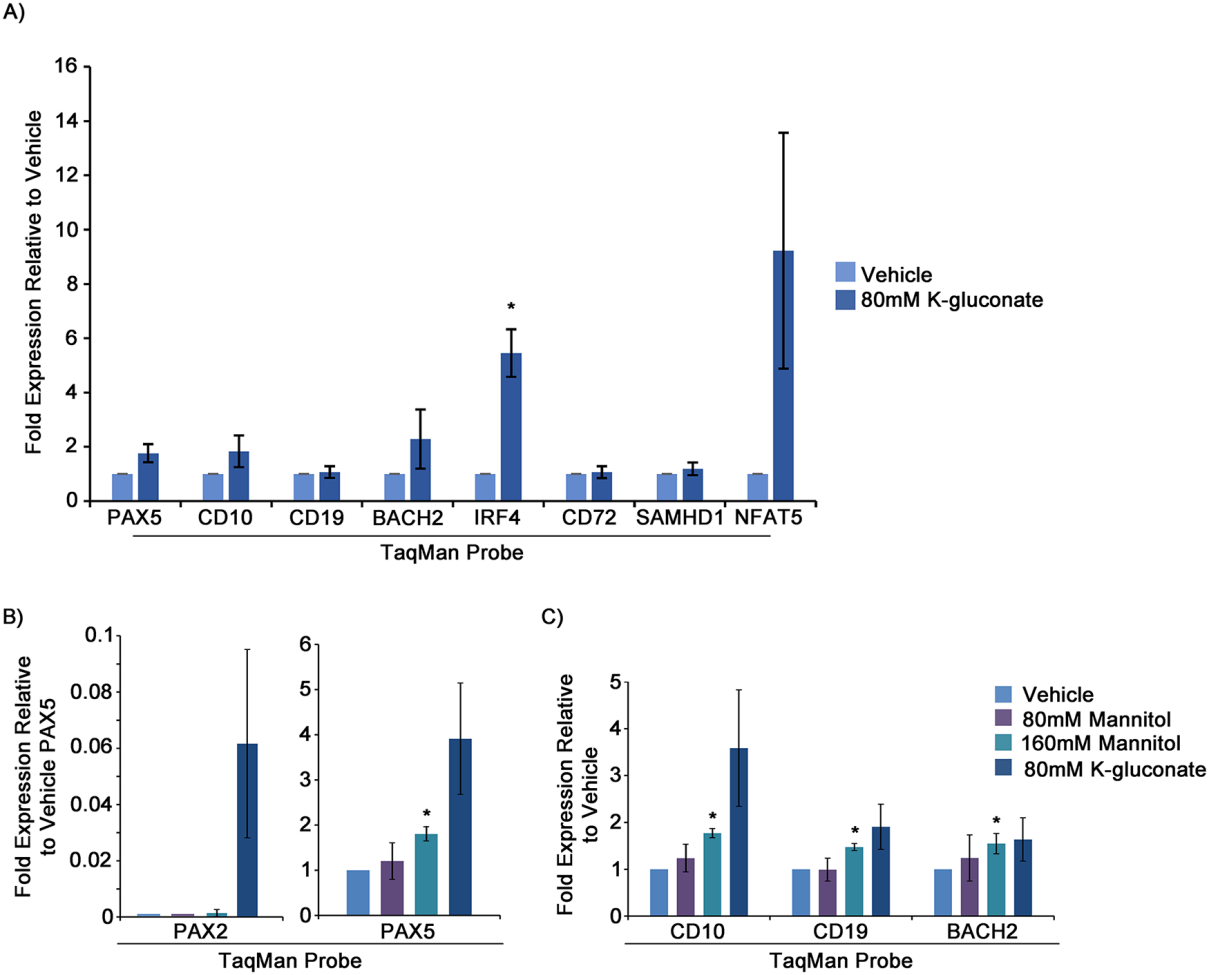
*PAX5* upregulation and downstream gene modulation in response to 80mM K-gluconate in a PAX5 mutant primary ALL sample, and cellular response to near clinical dosing of mannitol, support the potential of targeting hypertonicity response pathways in vivo. A) qRT-PCR analysis of *PAX2*, *PAX5*, and several downstream genes for NSGS mouse passaged aliquots of primary patient sample in response to 24 hour treatment with 80mM K-gluconate. Shown are two experimental replicates from separately thawed aliquots. Cells were sorted by FSC-A/SSC-A for live cells prior to isolation/harvest of RNA. B and C) qRT-PCR analysis of gene expression in Reh cells in response to 24 hour treatment with 80 or 160mM mannitol, compared with 80mM K-gluconate. Shown are 3 experimental replicates. Values are 2^-ΔΔC_T_^, relative to vehicle-treated. Statistical significance derived using one sample t-test vs. vehicle treated, assuming unequal variance (vehicle = 1), p-values * <0.05, ** <0.005, *** <0.0005. See also S14A Fig for a single replicate of a non-NSG passaged primary cell sample and S14B Fig for Reh viability in response to 80mM and 160mM mannitol vs. 80mM K-gluconate or CaCl_2_.

As numerous studies have shown, long term, in vitro, cell culture inherently selects for gene expression profiles differing from those seen for primary tissue samples [49,50]. To further evaluate whether the *PAX2*/*5* response to hyperosmolarity is one that is intrinsic to ALL cells both in vitro and in vivo, we screened 10 primary pre-B ALL samples for *PAX5* mutations, using Sanger DNA sequencing. Of those samples, one, from a 19 year-old male with trisomy 21 Down syndrome, possessed a heterozygous p.(K198Qfs*44) mutation, resulting in frameshift leading to early stop and protein truncation (see Methods). Pre-B ALL occurs more commonly in Down syndrome individuals and is felt to be biologically distinct from disease occurring in non-Down syndrome patients [51]; nevertheless, inactivating mutations of *PAX5* are detected at similar frequency in Down syndrome-associated pre-B ALL [52]. Due to limited sample availability from this patient, we performed a single test employing primary cells alongside multiple replicates using primary cells expanded through passage in mice (see Methods). Whether direct from the patient or passaged through mice, 24 hour exposure to 80mM K-gluconate resulted in increased expression of *PAX5*, as well as several but not all downstream targets seen previously with Reh and 697 cells (Fig 9A and S10A Fig). *PAX2* expression was not detected in this assay; however, this may be due in part to low RNA input levels, which were constrained by sample quantity.

The osmotic concentrations of K-gluconate or CaCl_2_ we evaluated in vitro would prove lethal if administered clinically. However, mannitol is also known to activate NFAT5 [53] and is used to adjust serum hyperosmolar concentrations to high levels in certain clinical settings [54]. To test whether mannitol could be employed to upregulate *PAX2* or *PAX5* in pre-B ALL, we treated Reh cells with 80mM or 160mM mannitol for 24 hours, prior to FACS sorting for live cells and harvesting of RNA. qRT-PCR demonstrated dose-dependent increases both for *PAX2* and especially for *PAX5*, along with similar changes in downstream gene expression, albeit not to the level seen with K-gluconate (Fig 9B and 9C). Importantly, 160mM is near the range of clinically achievable therapeutic concentrations for mannitol [54]. Comparison of 160mM mannitol with 80mM K-gluconate or CaCl_2_, followed by FSC-A/SSC-A sorting of live cells and subsequent measurement of culture expansion demonstrated slightly reduced growth potential for K-gluconate and CaCl_2_ treated cells as compared to cells grown in 160mM mannitol or normal media (S10B Fig). Interpretation of long term viability in response to hyperosmolarity was complicated due to the noticeably brief induction of *PAX2*/*5* (Fig 5E), coupled with the generally harsh nature of such treatment, even with only 24 hour exposure. The growth delay observed with K-gluconate in this case may largely be due to cell cycle arrest or other adverse effects of elevated hyperosmolarity [55], rather than the *PAX* dependent, developmentally programed exit from the cell cycle we appeared to observe with continuous *PAX* re-expression. However, even in vitro, mannitol appears to be better tolerated, and thus it or related organic osmolytes may present options for modulating tonicity that could prove tolerable in vivo.

## Discussion

Liu *et al*. recently demonstrated that restoration of *PAX5* expression can reverse the developmental blockade holding *PAX5*-mutated pre-B ALL cells in a continuously replicating, developmentally immature state [18]. We have confirmed that result and extended it further by showing that *PAX5*’s closely related paralogs, *PAX2* or *PAX8*, neither of which is mutated in ALL nor ordinarily expressed in lymphocytes, can function equivalently to normalize differentiation and growth of pre-B ALL cells. Moreover, we have shown that endogenous *PAX2* expression, and unexpectedly also *PAX5* itself, can be upregulated to promote similar effects on differentiation of pre-B ALL cells under hypertonic conditions.

While germline loss-of-function mutations are a cause of familial pre-B ALL [13,14], demonstrating that *PAX5* deficiency can ultimately initiate leukemogenesis, loss of *PAX5* activity is not by itself sufficient, and development of leukemia requires additional cooperating mutations. Cancer genome sequencing has identified a wide diversity of mutations [8,9], such that no two ALL patients are likely to share identical mutational profiles. Reh and 697 cells, tested here as well as by Liu *et al*. [18], are quite dissimilar, with 697 cells having only a few coding sequence alterations while Reh cells have considerably more, with very little overlap (S11 Fig). In particular, Reh cells contain a heterozygous loss-of-function *PAX5* mutation [33], whereas in 697 cells, *PAX5* is intact (per our sequencing, S13 Fig). However, an upstream regulator of *PAX5*, *E2A (TCF3)*, is at least partially inactivated via a translocation involving *PBX1* [35], suggesting that there may be similarly reduced expression of *PAX5* in 697 cells. Regardless, targeting *PAX2*/5/8 activity may prove beneficial even in those patients lacking *PAX5* mutations. Liu *et al*. also demonstrated that *PAX5* replenishment succeeded in curing a transgenic mouse model of ALL, driven by *PAX5* knockdown combined with *Stat5* activation [18]. The fact that *PAX5* re-expression normalizes growth and differentiation in pre-B ALL with divergent genetic backgrounds and mutational signatures, including with Down syndrome associated ALL as tested in primary cells, suggests that even after cooperating mutations have arisen, loss of *PAX5* activity continues to support the leukemic state. Consistent with the concept of oncogene addiction, in which secondary mutations are dependent upon driver mutations for maintaining the cancer phenotype [56], acquisition of additional mutations may therefore possibly render ALL cells even more vulnerable following replacement of *PAX5* activity.

Current approaches for treatment of pre-B ALL continue to rely on chemotherapy and, more recently, immunotherapy. Chemotherapy is often successful in pediatric settings [1] but is associated with considerable toxicity, long-term side effects [2], and substantially reduced efficacy in older children and adults, where allogenic stem cell transplant is more heavily relied upon [3]. Recent breakthroughs in CAR T cell therapy have shown great promise in treating certain disease presentations, specifically those which are highly CD19 positive [57]. However, hurdles remain, including clonal selection for *PAX5* deletion with consequent downregulation of the CD19 target antigen, leading to disease resistance [58]. Since CD19 is a direct target of *PAX5* and, as we have shown, can be activated equally well by *PAX2* or *PAX8*, the therapeutic approach contemplated here may work in conjunction with CAR T cell therapy by increasing levels of the targeted CD19 B cell antigen, even after loss of *PAX5*. CD19 can also be targeted through other forms of therapy, such as with antibody-drug conjugates [59].

Our observations demonstrate the use of gene paralogs to resolve a human disease phenotype. A remaining challenge, however, involves approaches for activating developmentally silenced genes in vivo. We tested a variety of compounds based upon previously described properties as either generally reversing repressive chromatin modifications (zebularine, hydralazine, valproic acid, azacitidine, and vorinostat) or as mechanistically undefined inducers of lymphocyte differentiation (ATRA, methotrexate, and phorbol 12-myristate 13-acetate (PMA)). None consistently activated *PAX2* or *PAX8* expression or otherwise promoted pre-B ALL cell differentiation under conditions we evaluated.

Another class of compounds we tested affect cell membrane potential, the modulation of which has been shown in model systems to induce a variety of developmental transcription factors, including, for example, *PAX6* [40]. We observed induction of *PAX2*, but not *PAX8*, as well as increases in downstream differentiation markers in response to K-gluconate. After testing a variety of other salts as well as several non-ionic modulators of cell membrane potential, we concluded that our observation was likely a cellular response to hypertonicity. During water diuresis, physiological concentrations of salts, mainly NaCl, in the renal inter-medullary interstitial fluid reach concentrations ranging from 600 to more than 1000mOsmol/kg H_2_O. Interestingly, survival mechanisms for cells in these conditions include the anti-apoptotic upregulation of *PAX2*, which has been shown to peak in mouse intermedullary collecting duct cells at ~500mOsmol/kg H_2_O [30], similar to what we observed in Reh cells (~400-540mOsmol/kg H_2_O in RPMI media). Unexpectedly, we observed that hypertonicity also induced expression of *PAX5* in pre-B ALL.

RNA-seq performed in conjunction with GSEA highlighted similarities and differences resulting from expression of *PAX2* or *PAX8*, compared to *PAX5*, in *PAX5*-deficient ALL cells. In a pairwise comparison of any of the three *PAX* factors, slightly fewer than half of all gene sets exhibiting significant expression changes were common to both, and only 13% of all gene sets (57/440, Fig 6A) enriched by *PAX5* were commonly modulated by all three *PAX* genes. Importantly, however, the group of gene sets commonly regulated by all three *PAX* factors includes *PAX5* targets most relevant to B cell maturation (Fig 6D), consistent with our findings that all three *PAX* factors similarly promote differentiation of *PAX5*-deficient ALL cells. We speculate that PAX5 target genes likely reside in accessible chromatin configurations in pre-B cells, such that even imperfect *PAX* activity from a paralog may readily induce their expression. In contrast, gene sets exhibiting significant enrichment following treatment with CaCl_2_ or K-gluconate exhibited much greater overlap with *PAX5*, and a majority of gene sets showing enrichment with *PAX5* (221/420, Fig 6B) or that were commonly enriched by all three *PAX* factors (43/57, Fig 6C) were also enriched after treatment with CaCl_2_ or K-gluconate. This may not be surprising given that treatment with either salt induced expression of *PAX2* and, especially, *PAX5* itself. Finally, it is worth emphasizing from a translationally relevant standpoint, that a set of 31 genes found by Liu et al. to undergo significant regulation during ALL remission, as induced by *Pax5* restoration in a mouse model of *Pax5*-deficient ALL, were similarly modulated by all tested conditions in our studies, whether it be *PAX5*, *PAX2*, *PAX8*, K-gluconate, or CaCl_2_ (Fig 6C).

We found that components of the *NFAT5* pathway, including *NFAT5 and TNFS13B (BAFF)*, along with its receptor, *TNFRSF13C (BAFF-R)*, are upregulated in response to many or all of our treatments (i.e., *PAX2*/*5*/*8* or salt treatment, Fig 8A and S1 Dataset). Named “nuclear factor of activated T cells 5,” for its role as a transcriptional coordinator of T cell immune response [60], *NFAT5* is the only known osmosensing mammalian transcription factor and is active in a variety of cell types, including B cells [45,46]. Indeed, siRNA mediated knockdown of *NFAT5* in Reh cells led to a reduction in *PAX2* expression in response to hyperosmolarity (Fig 8B and 8C). However, the added observation that *PAX5* expression was not affected by *NFAT5* knockdown suggests either the presence of a separate, non *NFAT5* related, osmosensing pathway upstream of *PAX5*, or alternatively, a substantially lower threshold for *NFAT5* abundance to achieve upregulation of *PAX5* under these conditions. In support of the latter, *PAX5* appears to contain more potential NFAT5 binding sites than *PAX2* (S9 Fig). Separately, these siRNA experiments showed that *PAX5* upregulation had a greater effect on downstream gene regulation, and presumably B cell maturation, than did *PAX2* (Fig 8B and 8C). Based on our observations from earlier experiments (Figs 2–4), where *PAX2* effectively functionally mimicked *PAX5*, and the substantially lower level of *PAX2* expression present relative to the induced levels of *PAX5* in response to hyperosmolarity (~20 fold), we believe this most likely reflects relative levels of expression, rather than differences in functionality.

Given that components of the hypertonicity response pathway are highly conserved from single cell organisms to mammals [61], it seems reasonable to speculate that *PAX* genes, including *PAX2* and *PAX5*, may play a role in osmotic adaptation across various tissue types. In fact, similar to our observations, upregulation of *PAX2* occurs in mouse embryonic fibroblasts in response to hypertonicity [48]. Secondary lymphoid organs, including spleen and thymus, maintain a remarkably high osmolar environment compared to serum and other tissue [62]. It should not be overlooked that a decrease in cell size, which we observed upon expression of *PAX2*/*5*/*8*, normally accompanies the large-to-small pre-B cell transition as cells begin their migration from the bone marrow to secondary lymphoid organs. It is possible that exposure to differences in local osmolarity across these compartments could play a role in normal lymphocyte development. Whether upregulation of *PAX2* and/or *PAX5* is a normal physiologic response to osmotic stress in lymphocytes or a vestigial pathway more heavily relied on in other tissues such as the kidney, but which is capable of artefactual activation under extreme circumstances, we show here that osmotic stress exposes a potential therapeutic target for activating elements of the normal B cell differentiation program.

The osmolar concentration required for peak induction of *PAX2* and *PAX5* is, just barely, outside the clinically achievable range for serum based on maximum recommended dosing for mannitol [54]. It is possible that specialized delivery methods, manipulation of dosage levels, and/or exposure time may bridge this gap. It is also worth noting that serum osmolar concentrations within this range are sometimes encountered in acutely ill diabetes mellitus patients with hyperglycemic hyperosmolar syndrome [63]. However, even if the highly hyperosmolar conditions we subjected ALL cells to in vitro are not therapeutically tenable in vivo, they do suggest that the complexity of kinases and other components of the signaling pathway responding to hypertonicity, including those regulating *NFAT5*, at least in the case of *PAX2* [64], may be ripe for investigation as drug targets.

An additional limitation relates to duration of therapy, as the replacement of *PAX5* activity may only have a temporary effect on differentiation of ALL cells, though this may still be beneficial either as a form of induction therapy or as an adjuvant when combined with CAR T cell, other therapies targeting CD19, or conventional chemotherapy. Intriguingly, a relevant recent in vitro study demonstrated that hyperosmotic stress achieved with salt or mannitol treatment synergized with chemotherapeutic drugs to kill ALL cells via an NFAT5 dependent mechanism, although activation of *PAX* genes was not investigated [53]. It should also be emphasized that remissions achieved with differentiation therapy employing ATRA for promyelocytic leukemia can actually be enduring [7]. Finally, if differentiation of pre-B ALL cells could be pushed as far as to the plasma cell stage, where *PAX5* expression is normally extinguished [65], then mutations inactivating *PAX5* could become inconsequential, anyway.

Finding the right balance of *PAX* gene expression is another issue. *PAX2*, when activated, can behave as an oncogene in solid tumors [66], and *PAX5* is normally down-regulated during plasma cell differentiation [65]. However, our RNA-seq data suggest that there may be an auto-regulatory ceiling for *PAX* gene expression, particularly for *PAX5*. Specifically, by examining total *PAX5* transcripts and comparing differences in the read ratios of SNPs discriminating between native and exogenous *PAX5*, we observed an apparent suppression of endogenous *PAX5* transcript by *PAX5* transgene expression, and to a lesser extent, by the expression of *PAX2* or *PAX8* transgenes (S12 Fig). Of course, unless *PAX* gene activation is confined only to the leukemic population of cells, there may be undesirable effects in other tissues, although compared to oncogenic mutations, *PAX* gene activation by osmoresponsive mechanisms is unlikely to be permanent. Moreover, some current cancer therapies employ treatment with epigenetic modifier drugs, such as azacitidine, capable of producing genome-wide and persistent activation of many genes across multiple tissues [67].

The strategy implemented here, to activate expression of intact and functionally similar paralogs of mutated cancer-driver genes to therapeutically restore cellular differentiation, could potentially be extended to other types of cancer. For example, inactivating *RUNX1* mutations frequently occur in acute myeloid leukemia, where upregulation of *RUNX2* or *RUNX3* exhibits anti-leukemic effects [68]. More generally, a wide variety of non-cancer illnesses possess etiologies for which complementation of inactivating mutations by activating gene paralogs may prove useful, extending the potential therapeutic application of this concept. For example, in spinal muscular atrophy, causative loss-of-function mutations in *SMN1* can be rescued by a recently approved therapy which uses an antisense compound to promote exon retention in an alternatively spliced yet otherwise identical paralog, *SMN2* [69]. Finally, hypertonic activation of *PAX* gene expression offers an example of emerging “electroceutical” approaches based on manipulation of biophysical phenomena [41].

## Methods

### Ethics statement

Leukemia cells were collected, after informed consent, through the Cell Bank of the Center for Cancer and Blood Disorders at Children’s Hospital Colorado. The Cell Bank protocol is approved by the Colorado Multiple Institutional Review Board (COMIRB #00–206).

Animal use was approved by the Animal Care and Use Committee of the University of Colorado Denver (Protocol 66912(12)1E).

### Contact for reagent and resource sharing

Further information and requests for resources and reagents should be directed to and will be fulfilled by the Lead Contact, Marshall Horwitz (horwitz@uw.edu).

### Experimental model and subject details

#### Cell lines and culture methods

Reh cells (female [32]) and HEK293T cells (female [70]) were obtained from ATCC. 697 cells (male [34]) were obtained from DSMZ. As suggested by their supplier, Reh and 697 cells were grown in 10% FBS/RPMI 1640 (Gibco, 11875–093), and HEK293T cells were grown in 10% FBS/DMEM (Gibco, 11995–065). All cells were grown at 37°C, under 5% CO_2_ and were passaged every 3–4 days at confluencies suggested by their suppliers. Cell aliquots were cultured for no more than ~20 passages. Based on hybrid capture exome sequencing, 697 cells are reported in the Cancer Cell Line Encyclopedia (CCLE) [71] as being heterozygous for the *PAX5* null mutation p.R225fs; however, we could not confirm this mutation upon Sanger DNA sequencing (S13 Fig), and review of the CCLE primary data shows that both read depth and variant allele count at this position are minimal, consistent with artifact. To verify the identity of the 697 cell line in our possession, we performed Sanger DNA sequencing and confirmed the existence of two separate and uncommon CCLE-reported gene mutations, intrinsic to this cell line; heterozygous *NRAS* p.G12D and hemizygous *GPR112* p.D2657del (S13 Fig). While not identified in the CCLE, Reh cells have been previously reported to contain a heterozygous p.A322fs mutation [33], which we confirmed by Sanger sequencing (S13 Fig). For further verification of cell line identity, low passage samples of both Reh and 697 cells were submitted to ATCC for short tandem repeat profiling and validation by comparison with known profiles for Reh and 697 cell lines. The samples were profiled using the PowerPlex 18D system (Promega), which provides a signature encompassing 17 short tandem repeat loci plus Amelogenin (S14 and S15 Figs).

### Primary patient samples

Ten B ALL samples were screened for mutation in *PAX5* by Sanger DNA sequencing. One sample, CHCO-7657, was found to contain a heterozygous *PAX5* mutation resulting in p.K198fs (S16 Fig). A subset of primary cells were stored in liquid nitrogen and additional leukemia cells were passaged through NOD *scid* gamma (NSG) mice once and amplified in NOD *scid* gamma Il3-GM-SF (NSGS) mice (both purchased from Jackson Labs), prior to storage in liquid nitrogen. Recipient mice were irradiated with 200cGy via X-irradiator prior to leukemia injection. Prior to experimentation, cells were thawed and resuspended in 20%FBS/MEM-alpha (Gibco,12561–056) which had been preconditioned with OP9 feeder cells (ATCC) seeded the day prior at 3×10^5^ cells/T75 flask, in 10mL media. Primary cells were overlaid onto and co-cultured with these OP9 feeder cells for 24 hours, followed by treatment with 80mM K-gluconate for an additional 24 hours before sorting for live cells and isolation of RNA.

### Method detail

#### Lentiviral plasmids cloning, virus preparation, and transduction

Lentiviral plasmids pRRL-CMV-IRES-hrGFPII and pLVX-EF1α-IRES-ZsGreen, were obtained from the University of Washington Diabetes Research Core and Clontech, respectively. *PAX5* isoform 1 cDNA [13] was utilized for these studies. *PAX2b* (catalog # SC300041) and *PAX8a* cDNA (SC122658) were obtained from OriGene. The *PAX2b* isoform was chosen due to its relatively abundant expression in human tissues and close resemblance to full length *PAX5* (S1 Fig). *PAX8a* is the most commonly expressed isoform [72] but includes an extra serine-rich ~60 amino acid region between the partial homeodomain and transactivation domain that is absent from *PAX2* or *PAX5*. EcoR1 sites were added to both ends of cDNA upon amplification with primers (PAX2F: 5’ACAGTAGAATTCGCCACCATGGATTACAAGGATGACGACGATAAGATGGATATGCACTGCAAAGCAGACC, PAX2R: 5’CTAGTGGCGGTCATAGGCAG, PAX8F: 5’ACAGTAGAATTCGCCACCATGGATTACAAGGATGACGACGATAAGATGCCTCACAACTCCATCAG, PAX8R: 5’CTACAGATGGTCAAAGGCCG) and were employed for ligation into plasmids. *PAX5*^p.V26fs^ was created via primer directed mutagenesis (Forward primer: 5’[Phos]GGGGTTTTTGTGAATGGACGG, Reverse primer: 5’[Phos]CCCCAAGCTGATTCACTCCTCC). Directionality and integrity were confirmed by Sanger sequencing. Expected protein size/expression was verified by western blot in HEK293T cells (visible in S3 Fig). Lentivirus production was performed by Allele Biotech and the University of Washington Diabetes Research Core Facility.

Cells were transduced using “spinoculation.” Briefly, cells were washed twice in serum-free RPMI and distributed at 2×10^5^ cells/well in a 96-well plate. Lentivirus (MOI 10–20) was added, along with 4.5 μg/mL polybrene for a total of ~100 μL volume/well. Cells were centrifuged at 1200×g at 30°C, for 2 hours. Supernatant was then removed and cells resuspended in a total of 1 mL growth media, then analyzed at day 4 post-transduction unless otherwise noted.

### Lysate preparation, SDS-PAGE and immunoblotting

Cells were lysed using RIPA buffer with complete protease inhibitor (Roche), 1mM Na_3_VO_4_ and 1mM PMSF. Lysates were quantified with the Pierce BCA Protein Assay Kit (ThermoFisher) and electrophoresed on MINI-protein TGX gels (BioRad) and transferred onto PVDF membrane (BioRad). Membranes were immunoblotted with indicated antibodies. All primary antibodies were diluted in 1% milk or BSA in TBST and were incubated overnight at 4°C. Blots were incubated in secondary antibody for 1 hour at room temperature, also in 1% milk or BSA.

### Flow cytometry and FACS

Flow cytometry for cell surface markers was performed on a BD LSR II flow cytometer using indicated antibodies. For antibody staining, cells were washed twice in sorting buffer (1%FBS/PBS), prior to incubation in antibody (diluted in sorting buffer) on ice and in the dark, for 30 minutes. Cells were then again washed twice in sorting buffer, and resuspended in 300–500μL sorting buffer prior to analysis. All staining and washing was done in 96 well, flat bottom plates. In between washes, cells were spun down for 3’ at 300×g. Plates were overturned and shaken to remove buffer. FACS was performed on a BD Aria II cell sorter. All raw data files were processed using FlowJo software. For experiments where RNA was harvested, cells were sorted directly into 500μL of Qiagen Buffer RLT+, prior to RNA isolation (see below). For further propagation of live cells, cells were sorted directly into complete growth media. Experiments were performed in triplicate (at minimum) unless otherwise noted in figure legends.

### RNA isolation and qRT-PCR

RNA was harvested from cells using the RNeasy Plus Mini Kit (Qiagen), following the supplied protocol, and converted to cDNA using random oligomers and either Superscript III or Superscript IV reverse transcriptase (Invitrogen). qRT-PCR was performed on cDNA using the indicated TaqMan probes and analyzed on an Applied Biosystems StepOnePlus Real-Time PCR System. Relative quantification of mRNA abundance was performed using the 2^-ΔΔC^_T_ method and *ACTB* or *GAPDH* as reference genes, where ΔC_T_ = (CT_target_-CT_reference_) and 2^-ΔΔC^_T_ = 2^-(ΔC^_Tsample_^-ΔC^_Tcontrol_^)^. Note, in the cases of *PAX2* and *PAX8*, for which no endogenous baseline expression was detected in ALL cells, baseline *PAX5* (empty vector or vehicle) expression was used in calculating ΔCT_control_. Experiments were performed in triplicate unless otherwise noted in figure legends.

### Cellular proliferation assays

At day 4 post-lentiviral transduction, 2×10^4^ ZsGreen-positive cells of each *PAX* gene or control vector type were isolated by FACS and distributed into individual single wells of a 96-well plate. Beginning with normalized concentrations of 2×10^5^ cells/mL (i.e., 100 μL total volume/well), these cells were further propagated in culture for a time course of 15–17 days. Culture density was assessed manually every 1–2 days during this time, using a hemocytometer. Additional media was added as needed prior to each counting in order to account for evaporation and to maintain ~100 μL volume in each well. For HEK293T cells, cell viability was assessed using an MTS assay (Cell Titer 96 One Solution, Promega), which produces a formazan product in the presence of phenazine methosulfate, which is present in metabolically active cells. Soluble formazan product is detectable at a 490nm absorbance maximum in PBS. Experiments were performed in triplicate (at minimum) unless otherwise noted in figure legends.

### Exposure to hypertonicity

Cells were passaged one day prior to plating at a density of 2×10^5^ cells/mL in 5 or 10mL of regular growth media with an added 80mM K-gluconate (Sigma, P1847) or CaCl_2_ (Sigma, C-3306) (unless otherwise noted). After indicated incubation times and depending on the experiment, RNA was either bulk harvested from treated cells or was harvested from live cells that were first sorted and collected by flow cytometry based on FSC-A and SCC-A measurements (indicated in figure legends). For pulse/chase in Fig 5E, 5F and 5G, cells were treated as indicated for 24 hours. 3×10^5^ live cells were then sorted and returned to culture followed by removal of aliquots at indicated time points for harvesting of RNA (0h = 24 hour pulse, 0 hour chase). Experiments for all figures were performed in triplicate (at minimum) unless otherwise noted in figure legends.

### siRNA

4×10^6^ Reh cells were electroporated with SMARTpool siRNA (i.e., 3 separate target siRNAs each) for *PAX2*, *PAX5*, *NFAT5*, or a non-targeting control pool (Dharmacon) using a BioRad GenePulser Xcell (Square wave, 210V, 15ms, 2x pulse, 0.1sec gap). Cells were suspended in 400μL Opti-MEM buffer containing 500nM siRNA that had been previously prepared and frozen at 20μM stock concentration in siRNA resuspension buffer (GE Healthcare). Cells were allowed to recover for 24 hours prior to harvest of protein lysates or treatment with hypertonic media (80mM K-Gluconate in RPMI with 10% FBS).

### RNA-seq

#### Transfection of *PAX* family members

Two separate transfections of *PAX2*, *PAX5*, *PAX8*, or empty vector (pRRL-CMV-IRES-hrGFPII) control were performed via electroporation in 400μL OPTI-MEM on 1.5×10^6^ Reh cells per treatment condition (BioRad GenePulser Xcell, Square wave, 210V, 15ms, 2x pulse, 0.1sec gap). The transfected cells were incubated for 24 hours post transfection in normal growth media (see above). Cells were washed twice in PBS plus 1% FBS, and 4–6×10^5^ GFP (+) cells per treatment condition were harvested via FACS for RNA. Total RNA was isolated from sorted cells using the Qiagen RNeasy Plus kit.

#### Salt treatment

1×10^6^ Reh cells, cultured in RPMI supplemented with 10% FBS, were exposed to either K-gluconate or CaCl_2_ (80mM concentration added to media) for 24 hours. The cells were then washed twice with PBS, and total RNA was isolated using the Qiagen RNeasy Plus kit. Each condition, along with a no treatment control, was repeated for a total of 3 samples.

#### Sequencing

mRNA libraries were prepared from total RNA (~1 μg per sample) using the Illumina TruSeq Stranded mRNA Library Prep kit. Individual RNA samples were created by pooling equal amounts of total RNA from multiple experiments (see above). Prepared libraries were sequenced on an Illumina NextSeq 500 instrument (1×75 bp read). Reads were aligned against GRCh38 using the HISAT2 aligner. Reads mapped to exons defined by GRCh38.88 were counted, and transcripts per million (TPM) values for each gene were calculated using StringTie software (Johns Hopkins Center for Computational Biology). An average of 53 million reads were aligned per sample.

### Quantification and statistical analysis

#### qRT-PCR

Bar graphs represent combined mean values of 2^-ΔΔCT^ for included experimental replicates, relative to controls indicated in the figure legend and graph axis labels. Error bars represent standard deviation. Significance was determined using one-way t-test method for deviation from a fixed value (i.e., value of control sample). p-values * <0.05, ** <0.005, *** <0.0005.

### Flow cytometry

Bar graphs for antibodies and cell size (FSC-A) represent mean fluorescence intensity. Values are averaged across several experimental replicates, as indicated, above. Error bars represent standard deviation. Significance was determined using one-way t-test method for deviation from a fixed value (i.e., normalized value of control sample). p-values * <0.05, ** <0.005, *** <0.0005. Bar graphs for cell cycle phase (S5 Fig) were determined from percentages of cells in G1, S, and G2 phase, based on DAPI staining, and assessed by the “Cell cycle” function in FlowJo, vX.

### RNA-seq

Normalization and differential expression calculations were performed using the R package DESeq2 [73] based on TPM data. Clustering and heatmap creation were performed using the heatmap.2 package (dist = Euclidean and method = complete).

### Gene set enrichment analysis (GSEA)

Expressed genes in each sample were ranked based on their log_2_ fold change in mRNA levels when compared to the appropriate control. GSEA was conducted using GSEA Desktop 3.0 software (Broad Institute). Gene sets analyzed (Molecular Signatures Database v6.1) include the biological process group from Gene Ontology (GO:BP), the transcription factor targets (TFT) group, and a custom *PAX5* related group of human genes based on genes differentially expressed at various stages of B cell development in mice that have either normal levels of *PAX5* or are deficient [13,43]. Briefly, the pro and mature B cell sets are comprised of genes that are differentially expressed compared to the appropriate controls and have predicted PAX5 binding sites in their promoter region while the pro-to-mature B cell sets contain all genes differentially expressed when comparing mature B cells to pro-B cells in the presence or absence of *PAX5*. Analysis was conducted using the GSEAPreranked tool to calculate a classic enrichment score for each set. Gene sets with a false discovery q-value (FDR) of <0.05 were selected for further analysis.

## Supporting information

S1 Fig *PAX2*/*5*/*8* domains share high levels of homology. Aligned amino acid sequences of functional domains for human *PAX2*, *PAX5*, and *PAX8* are shown. Homology to *PAX5* is illustrated at right, with percent identity as well as percent similarity listed for each domain.
(TIF)

S2 Fig Gating strategy for isolating single and/or ZsGreen positive cells. Sequential gating using FSC-A vs. SSC-A along with FSC-W vs. FSC-H and SSC-W vs. SSC-H allows for the isolation of single, largely viable cells. Further gating by presence or absence of GFP allows for isolation of lentivirally transduced cells, if applicable.
(TIF)

S3 Fig *PAX2/5/8* modulated cell viability and size changes are B cell specific. A) 293T cells were transfected with *PAX* lentiviral vectors using Lipofectamine 3000 following manufacturer suggestions. Protein lysates were taken at 24 hours, quantified by BCA protein assay, and analyzed by SDS-PAGE followed by western blot with anti-Flag antibody (BioLegend). Predicted size of *PAX5*^p.V26fs^ is roughly 8.7kDa (80aa), but was not detected by western blot, presumably due to complete nonsense-mediated decay. B) 6×10^4^ cells of each group were sorted by FACS for ZsGreen at day 4 post transduction with lentivirus expressing either *PAX* genes or *PAX5*^p.V26fs^ or empty vector (ZsGreen only) controls. Cells of the (-) ZsGreen sample are unsuccessfully transduced cells of the *PAX5* sample, as in Fig 4. Post sorting, the cells of each group were divided equally into 9 separate wells in a 96 well plate (~6.6×10^3^ cells/well). At 0, 4 and 6 days post sorting, 3 wells of each group were used to assess viability with an MTS colorimetric assay as described in the Methods. C) Histogram comparisons of cell size by FSC-A for 293T cells transduced with either *PAX2*/5/8, empty vector, or *PAX5*^p.V26fs^.
(TIF)

S4 Fig *PAX2* and *PAX8* promote exit from the cell cycle in *PAX5*-deficient pre-B ALL cells, additional data related to Fig 4. A) Reh (3 experimental replicates) and B) 697 (2 experimental replicates) cell culture density vs. time, following sorting (day 4 post transduction) for ZsGreen-positive cells expressing indicated transgenes. Data points for all replicates are shown, along with lines fitting the mean values for each treatment. Numbers for mean and standard deviation for all time points and treatments are shown below. (-) ZsGreen cells represent unsuccessfully transduced cells sorted from the *PAX5* lentivirus exposed cell suspension. C) Numerical representation of percentage of ZsGreen positive vs. negative cells at 11–14 days post sort for ZsGreen for 2 experimental replicates for per cell line.
(TIF)

S5 Fig *PAX2*/*5*/*8* expression leads to cell cycle delays and a modest increase in apoptosis. A) Cells were transduced with lentiviral *PAX* expression constructs as described (Methods) and sorted for ZsGreen at day 4 post transduction. Cells were immediately fixed and stained with DAPI, followed by flow analysis for staining intensity. Curves representing phases of the cell cycle were fitted using the “Cell Cycle” function of FlowJo software. Figure represents a single experimental replicate. B) Graphical representation of % cells per phase, based on the analysis in A. C) Reh cells were electroporated with either *PAX5* or empty vector expression constructs. 24 hours later, cells were stained with Annexin V/DAPI and analyzed by flow cytometry using the gating strategy shown.
(TIF)

S6 Fig Exposure to hyperosmolarity causes expression of *PAX2* and upregulation of *PAX5* in Reh cells. A-C) Cells were incubated for 24 hours with vehicle (normal growth media), media with added 80mM K-gluconate, or media with added 80mM CaCl_2_. RNA was then bulk harvested and cDNA prepared as described in the Methods. Representative *PAX2* (red) as well as *PAX5* (yellow) amplification curves are shown for all samples.
(TIF)

S7 Fig qRT-PCR normalization using *GAPDH* is similar to *ACTB* in Reh cells and 697 cells also respond to hypertonicity. A, B) Dose curve as in Fig 5C and 5D, except normalized to *GAPDH* rather than *ACTB*. C, D) Dose curve for K-gluconate treated 697 cells, normalized to *ACTB*. Note, both A and B represent an average of two experimental replicates.
(TIF)

S8 Fig qRT-PCR validation of RNA-seq gene regulation trends and *NFAT5* knockdown affects solute carrier upregulation in response to hyperosmolarity. A) qRT-PCR validation of RNA-seq gene subset from Fig 8. Fold change values are 2^-ΔΔC_T_^, relative to each samples’ respective control (i.e., empty vector or untreated), with *ACTB* used as endogenous reference gene. Represents 2 experimental replicates. B) Fold expression of solute channels (+/-) 80mM K-gluconate and (+/-) siRNA knockdown of *NFAT5* or *GAPDH* as a negative control. Represents 3 experimental replicates.
(TIF)

S9 Fig *PAX2* and *PAX5* genomic loci contain multiple TonE binding elements. A) Screen shot from UCSC Genome Browser image of the *PAX5* locus, highlighting instances of the TonE consensus sequence (TGAAANNYNY) which are present in the genomic region shown. B) As in A, but for the *PAX2*.
(TIF)

S10 Fig Exposure to hyperosmolarity results in *PAX5* and downstream gene modulation in a non-NSG passaged, *PAX5* mutant, primary patient pre-B ALL sample and has varied effects on cell viability in Reh cells. A) qRT-PCR analysis of *PAX2*, *PAX5*, and several downstream genes for one aliquot of direct-from-patient, primary sample in response to 24 hour treatment with 80mM K-gluconate. Cells were sorted by FSC-A/SSC-A for live cells prior to isolation/harvest of RNA. B) Reh cells were treated with 80 or 160mM mannitol, 80mM K-gluconate, or vehicle control for 24 hours, followed by FSC-A/SSC-A sorting for 2×10^5^ live cells per condition which were then return to culture. Culture density as shown, was evaluated manually at days 2, 3, and 5 post sort. Data points for 3 experimental replicates are shown, as are lines representing mean values of combined replicates.
(TIF)

S11 Fig Reh and 697 cell lines show disparate mutational profiles. Shown is a Venn diagram of all coding mutations listed in the CCLE for both 697 (black) and Reh cells (magenta). Note, while the CCLE shows a *PAX5* mutation in 697 cells, it is not included here as we did not detect that mutation by Sanger sequencing (see S13 Fig). However, we did confirm 697 identity by verifying the presence of other mutations and by short tandem repeat profiling (S14 Fig). Additionally, the p.A322fs *PAX5* mutation in Reh cells is not reported by the CCLE, but is shown here (bold), as it has been reported by other sources, and we have verified it by Sanger sequencing (see Methods, S13 Fig).
(TIF)

S12 Fig Exogenous *PAX* paralog expression reduces endogenous *PAX5* expression. A) Normalized RNA-seq expression data of PAX2/5/8 in transfected Reh cells. rlog normalized TPM values shown in cells transfected with pRRL- empty vector, *PAX2*, *PAX5*, or *PAX8*. Dark purple box indicates proportion of reads from Reh p.A322fs allele. B) *PAX5* variant sequences in Reh cells and pRLL-PAX5 cDNA. (+) strand genomic sequence is shown for exon 8 and 10 for both Reh alleles as well as pRLL-PAX5. Variants are shown in red. C) Proportion of PAX5 aligned reads attributable to either the Reh alleles or the pRLL-PAX5. For Exon 8: insG, the ratio of reads with an insertion to total reads in the empty vector sample from A was used to estimate the percentage of reads attributable to the Reh alleles. Black boxes indicate the range of Reh *PAX5* expected based on samples in A. Average read depth (SD) for each condition; Empty Vector = 570(65), PAX5 = 11×10^3^ (2.1×10^3^). Exon 8: T>C = NC_000009.12:g.36,882,065T>C, Exon 8: insG = NC_000009.12:g.36,882,053insG, Exon 10: G>A = NC_000009.12:g.36,840,626G>A.
(TIF)

S13 Fig Sanger DNA sequencing verification of Reh and 697 cell lines. Arrows denote the locations of previously reported mutations for Reh and 697 cells. A) Electropherogram from Sanger DNA sequencing of exon 8, Reh cells, showing a heterozygous single nucleotide insertion resulting in the p.A322fs mutation [33]. B) Electropherogram trace from exon 6, 697 cells, at the location of the CCLE identified p.R225fs mutation [71]. C) Electropherogram traces showing the presence of *GPR112* and *NRAS* mutations (p.D2657del and pG12D) as reported in the CCLE for the 697 cell line. Note that *GPR112* resides on the X chromosome, and the cell source is male.
(TIF)

S14 Fig Short tandem repeat profiling to validate identity of the Reh cell line. A) Electropherogram results for the Reh sample. B) Profile comparisons for the submitted sample and ATCC CRL-8286 (Reh cell line) for a subset of 18 evaluated loci.
(TIF)

S15 Fig Short tandem repeat profiling to validate identity of the 697 cell line. A) Electropherogram results for the 697 sample. B) Profile comparisons for the submitted sample and DSMZ-42 (697 cell line) for a subset of 18 evaluated loci.
(TIF)

S16 Fig Sanger DNA sequencing of primary patient sample. Forward and reverse Sanger traces show position of the p.K198fs mutation in *PAX5*.
(TIF)

S1 Table GSEA results. (XLSX)

S2 Table Custom gene sets. (XLSX)

S3 Table Leading edge genes for MYCMAX_01 and E2F1_Q4 gene sets. (XLSX)

S1 Dataset Exogenous *PAX2*/*5*/*8* expression or elevated hyperosmolarity modulate Reh gene expression. Excel file including log_2_ gene expression changes from treatment with *PAX2*/*5*/*8* vs. empty vector or treatment with 80mM K-gluconate or CaCl_2_ vs. untreated Reh cells (“Differential Expression” tab). Genes are listed by Ensembl ID. Treatment conditions used are outlined in the “Conditions Key” tab and described in the methods.
(XLSX)

S2 Dataset Numerical data underlying all graphical representations presented in main body and supplemental figures. Tabs are labeled with corresponding figure numbers. Corresponding sub-figure labels within each tab are highlighted in yellow.
(XLSX)
