# Supplementary figures and images for: Activating PAX gene family paralogs to complement PAX5 leukemia driver mutations
Source: PLoS Genet. 2018 Sep 14;14(9):e1007642. doi: 10.1371/journal.pgen.1007642 (PMC6157899; doi:10.1371/journal.pgen.1007642)

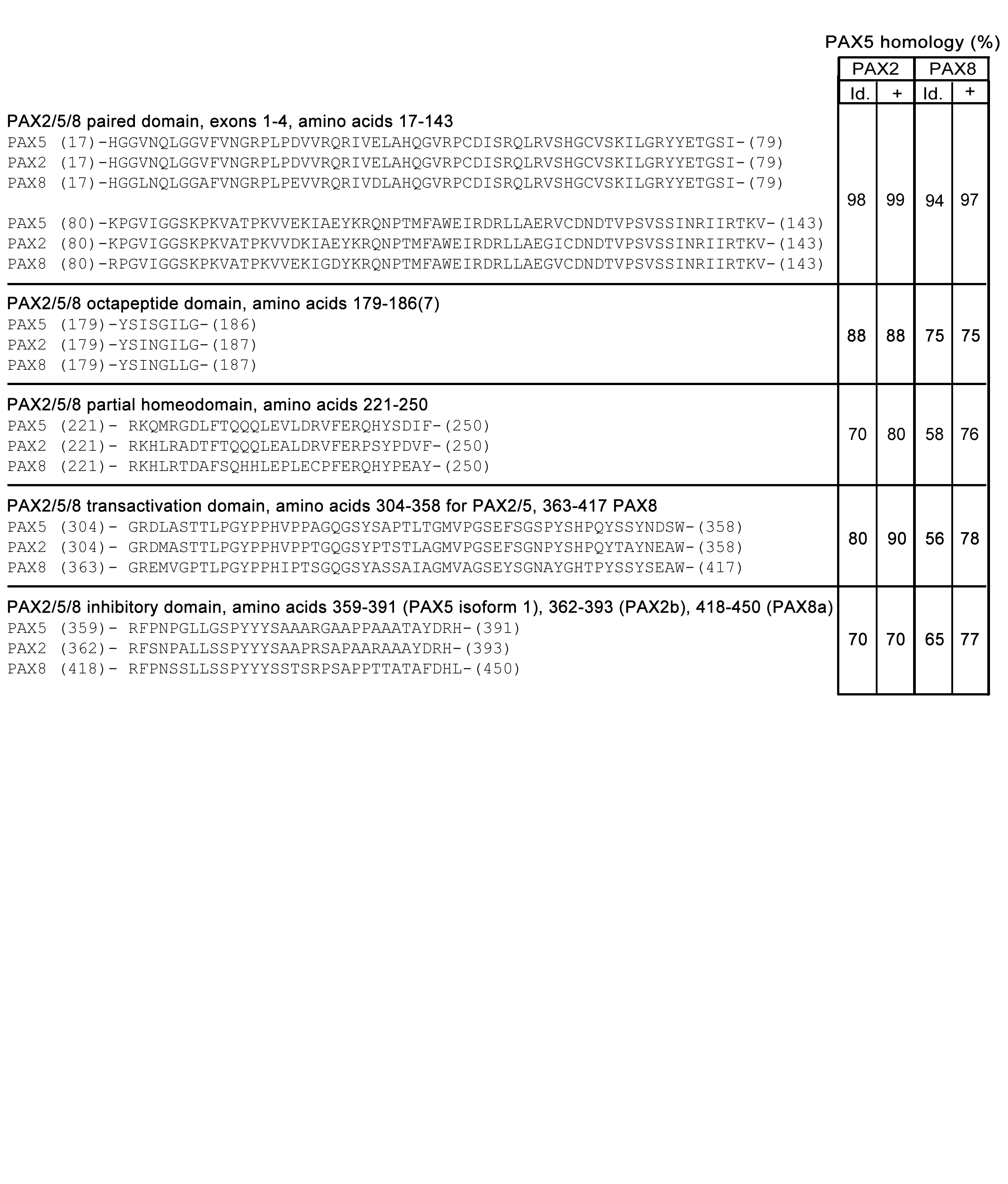

Supplement: S1 Fig — Aligned amino acid sequences of functional domains for human PAX2, PAX5, and PAX8 are shown. Homology to PAX5 is illustrated at right, with percent identity as well as percent similarity listed for each domain. (TIF) [file pgen.1007642.s001.tif]

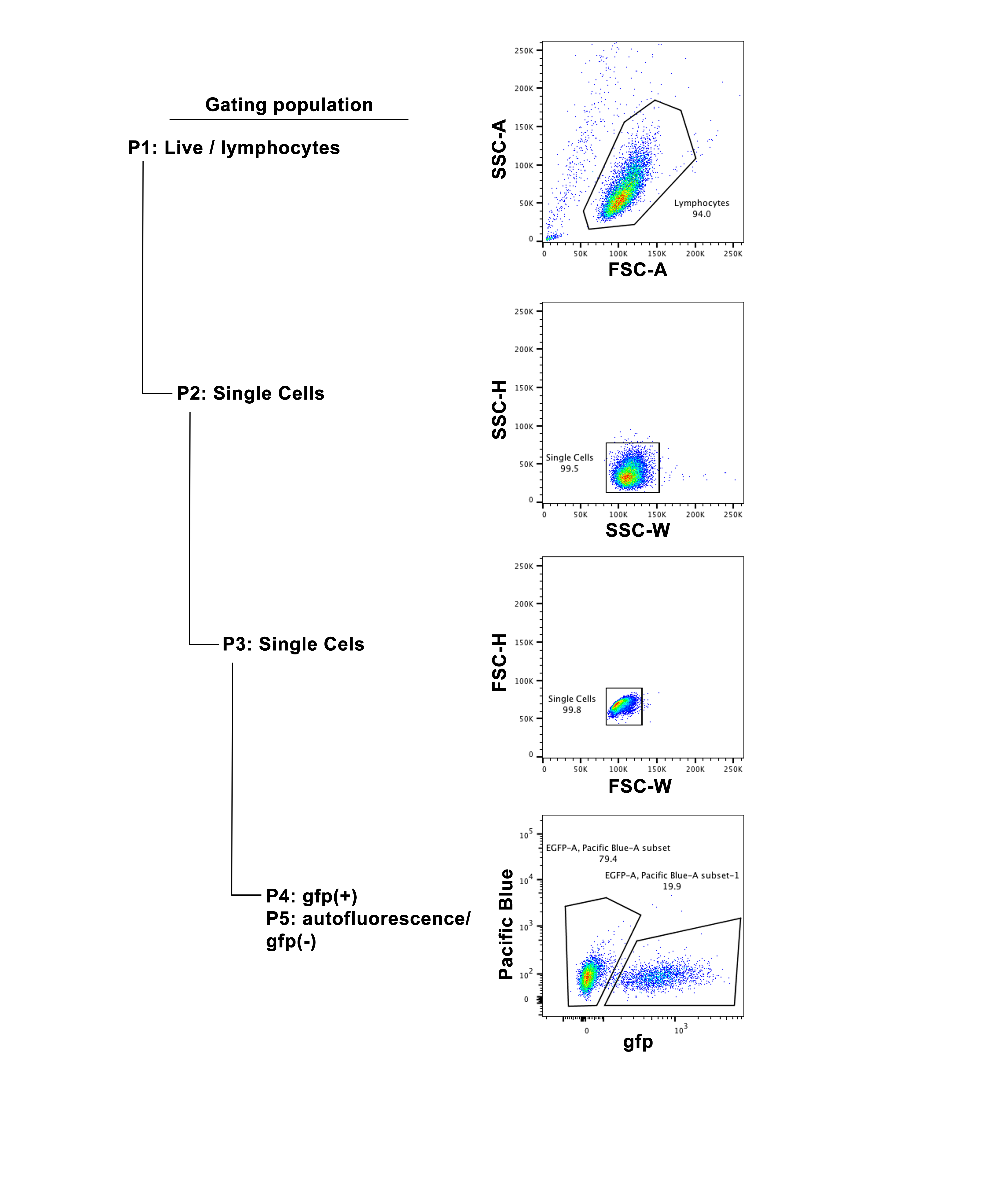

Supplement: S2 Fig — Sequential gating using FSC-A vs. SSC-A along with FSC-W vs. FSC-H and SSC-W vs. SSC-H allows for the isolation of single, largely viable cells. Further gating by presence or absence of GFP allows for isolation of lentivirally transduced cells, if applicable. (TIF) [file pgen.1007642.s002.tif]

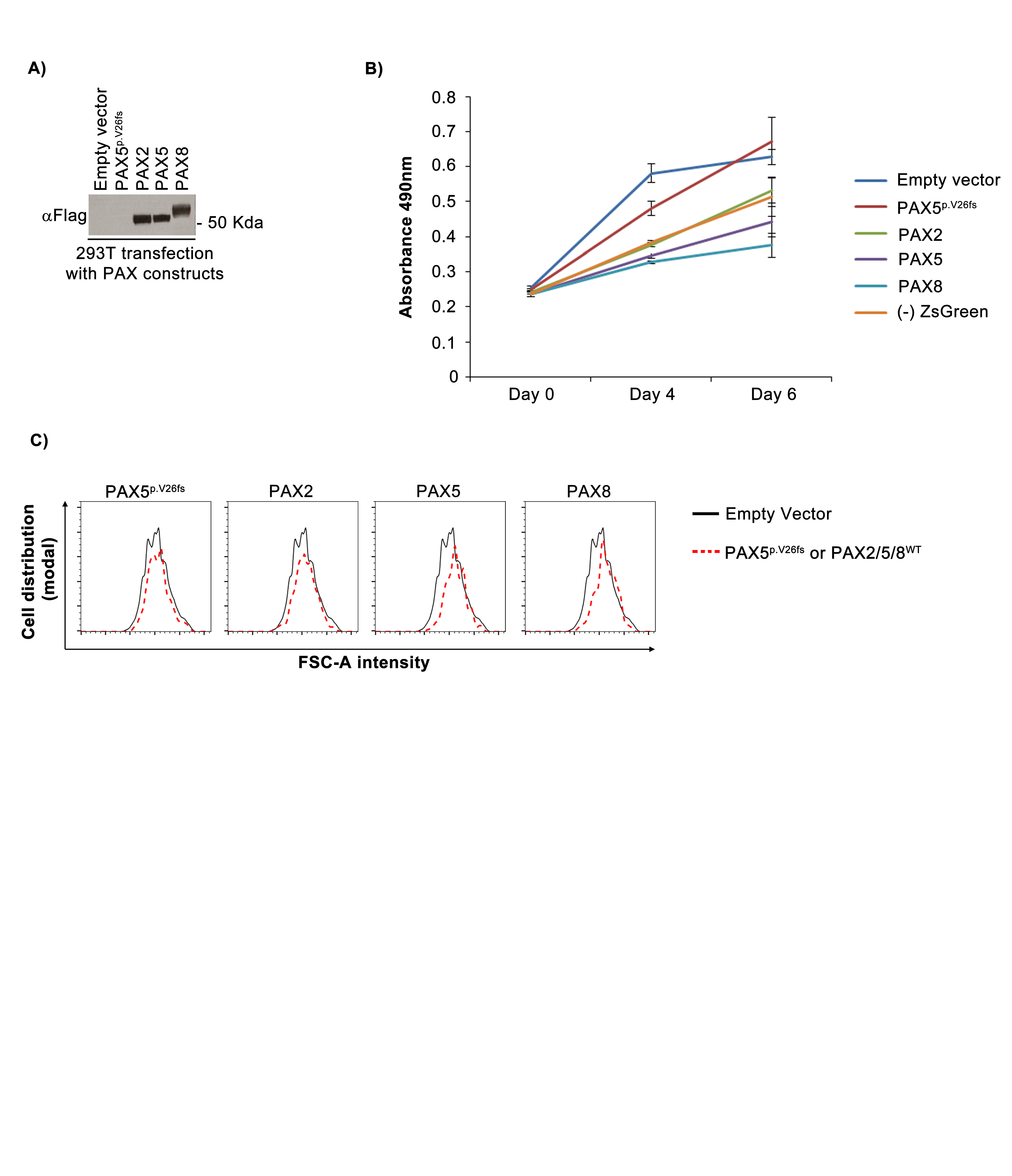

Supplement: S3 Fig — A) 293T cells were transfected with PAX lentiviral vectors using Lipofectamine 3000 following manufacturer suggestions. Protein lysates were taken at 24 hours, quantified by BCA protein assay, and analyzed by SDS-PAGE followed by western blot with anti-Flag antibody (BioLegend). Predicted size of PAX5p.V26fs is roughly 8.7kDa (80aa), but was not detected by western blot, presumably due to complete nonsense-mediated decay. B) 6×10^4^ cells of each group were sorted by FACS for ZsGreen at day 4 post transduction with lentivirus expressing either PAX genes or PAX5p.V26fs or empty vector (ZsGreen only) controls. Cells of the (-) ZsGreen sample are unsuccessfully transduced cells of the PAX5 sample, as in Fig 4. Post sorting, the cells of each group were divided equally into 9 separate wells in a 96 well plate (~6.6×10^3^ cells/well). At 0, 4 and 6 days post sorting, 3 wells of each group were used to assess viability with an MTS colorimetric assay as described in the Methods. C) Histogram comparisons of cell size by FSC-A for 293T cells transduced with either PAX2/5/8, empty vector, or PAX5p.V26fs. (TIF) [file pgen.1007642.s003.tif]

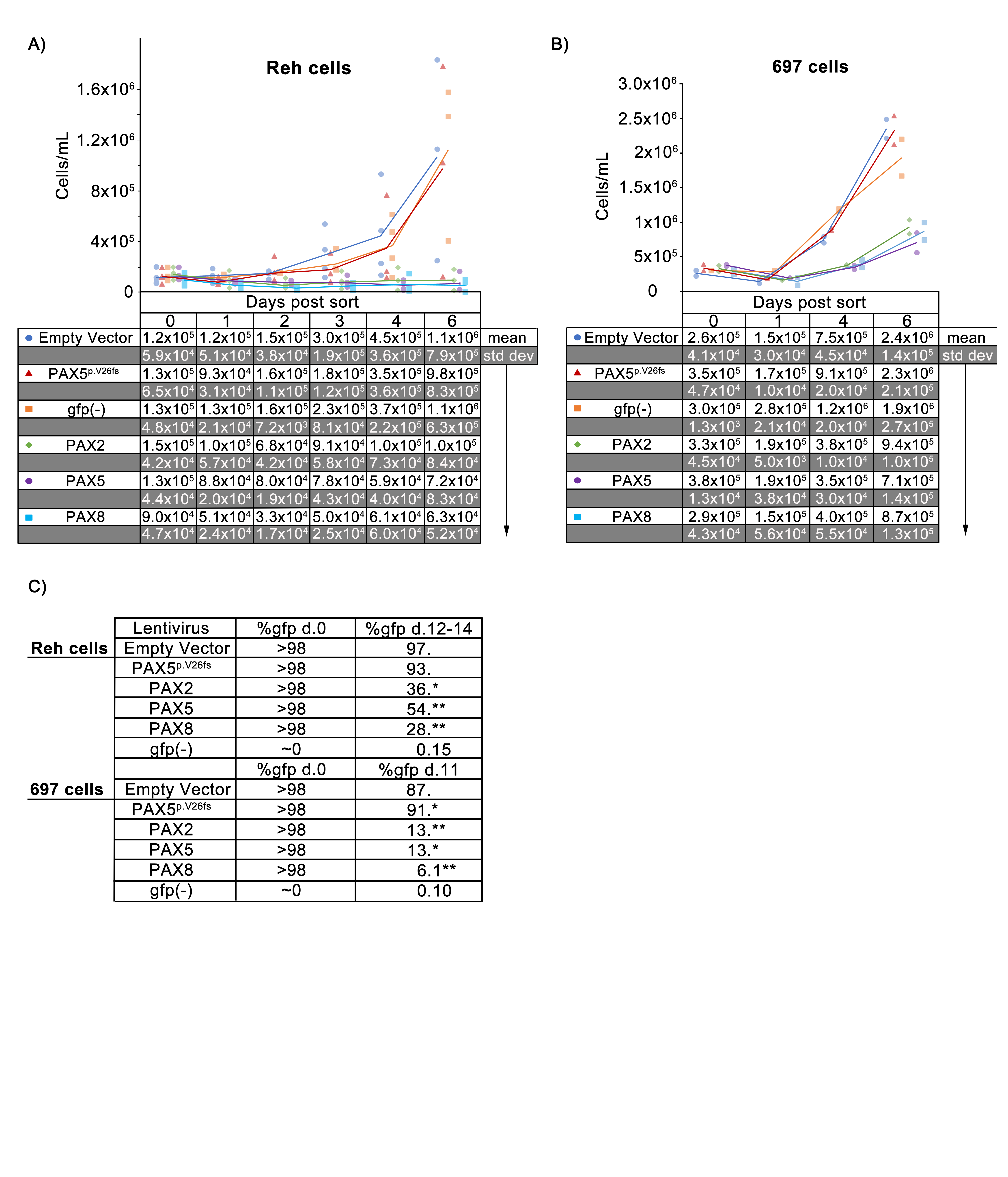

Supplement: S4 Fig — A) Reh (3 experimental replicates) and B) 697 (2 experimental replicates) cell culture density vs. time, following sorting (day 4 post transduction) for ZsGreen-positive cells expressing indicated transgenes. Data points for all replicates are shown, along with lines fitting the mean values for each treatment. Numbers for mean and standard deviation for all time points and treatments are shown below. (-) ZsGreen cells represent unsuccessfully transduced cells sorted from the PAX5 lentivirus exposed cell suspension. C) Numerical representation of percentage of ZsGreen positive vs. negative cells at 11–14 days post sort for ZsGreen for 2 experimental replicates for per cell line. (TIF) [file pgen.1007642.s004.tif]

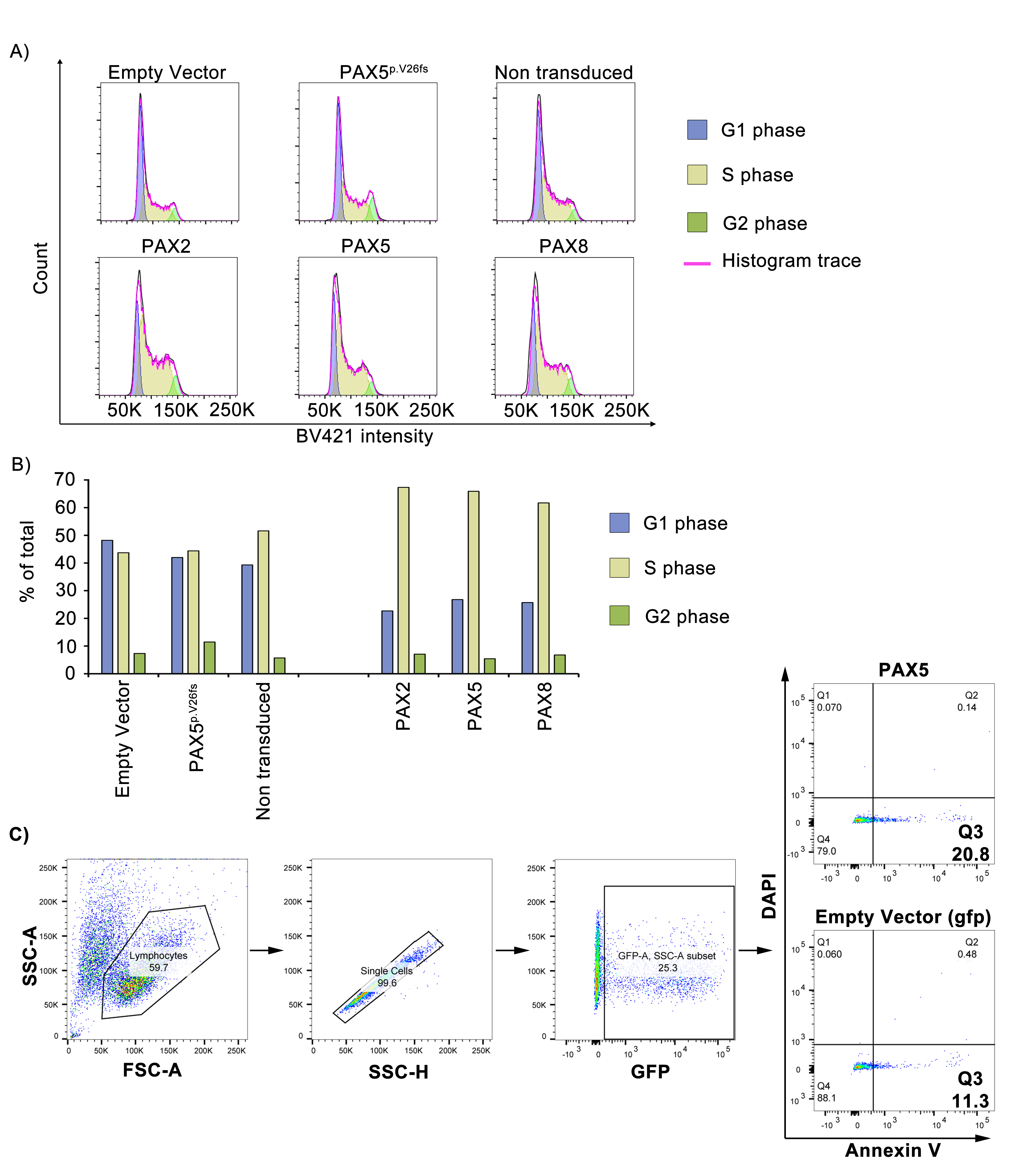

Supplement: S5 Fig — A) Cells were transduced with lentiviral PAX expression constructs as described (Methods) and sorted for ZsGreen at day 4 post transduction. Cells were immediately fixed and stained with DAPI, followed by flow analysis for staining intensity. Curves representing phases of the cell cycle were fitted using the “Cell Cycle” function of FlowJo software. Figure represents a single experimental replicate. B) Graphical representation of % cells per phase, based on the analysis in A. C) Reh cells were electroporated with either PAX5 or empty vector expression constructs. 24 hours later, cells were stained with Annexin V/DAPI and analyzed by flow cytometry using the gating strategy shown. (TIF) [file pgen.1007642.s005.tif]

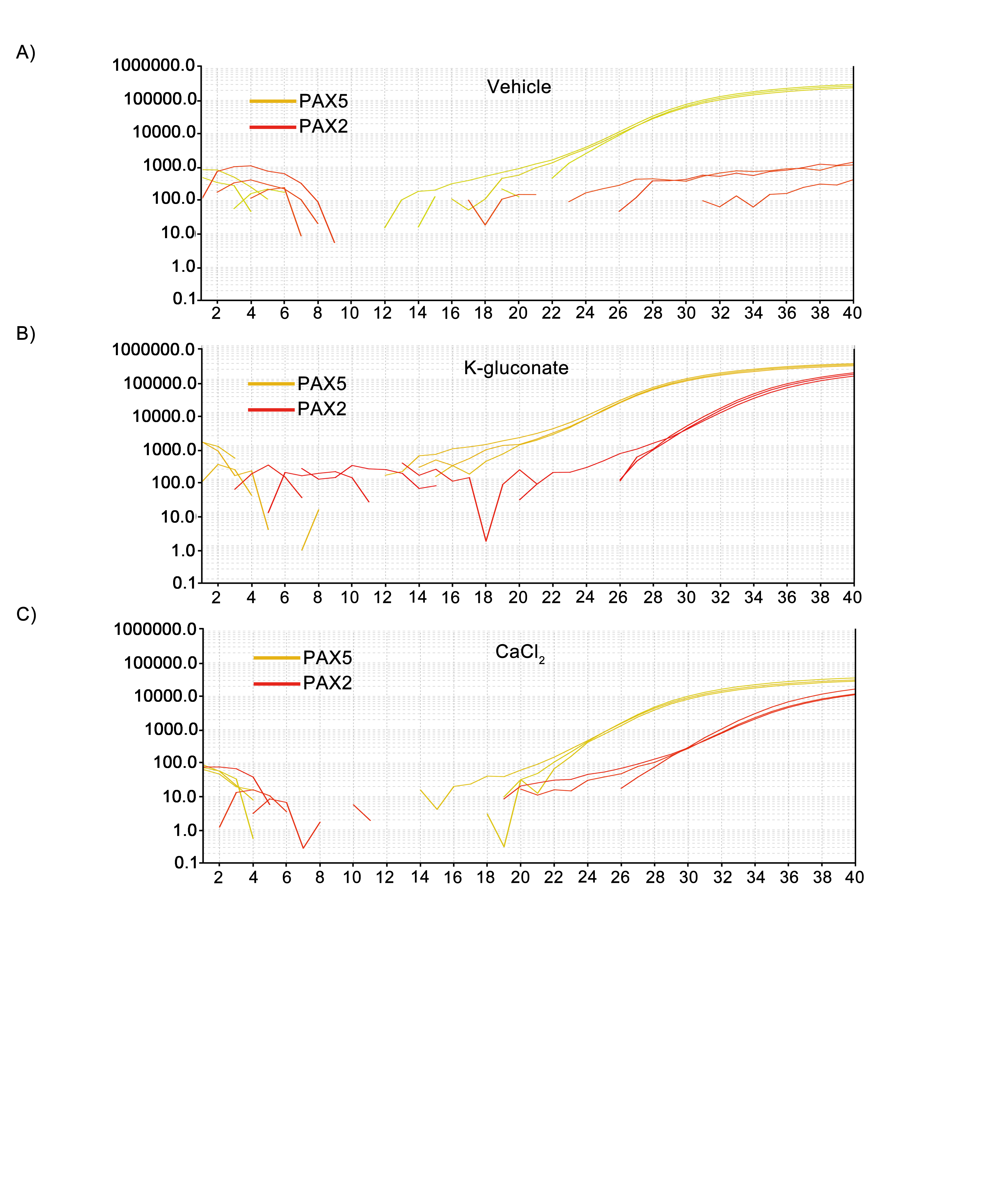

Supplement: S6 Fig — A-C) Cells were incubated for 24 hours with vehicle (normal growth media), media with added 80mM K-gluconate, or media with added 80mM CaCl2. RNA was then bulk harvested and cDNA prepared as described in the Methods. Representative PAX2 (red) as well as PAX5 (yellow) amplification curves are shown for all samples. (TIF) [file pgen.1007642.s006.tif]

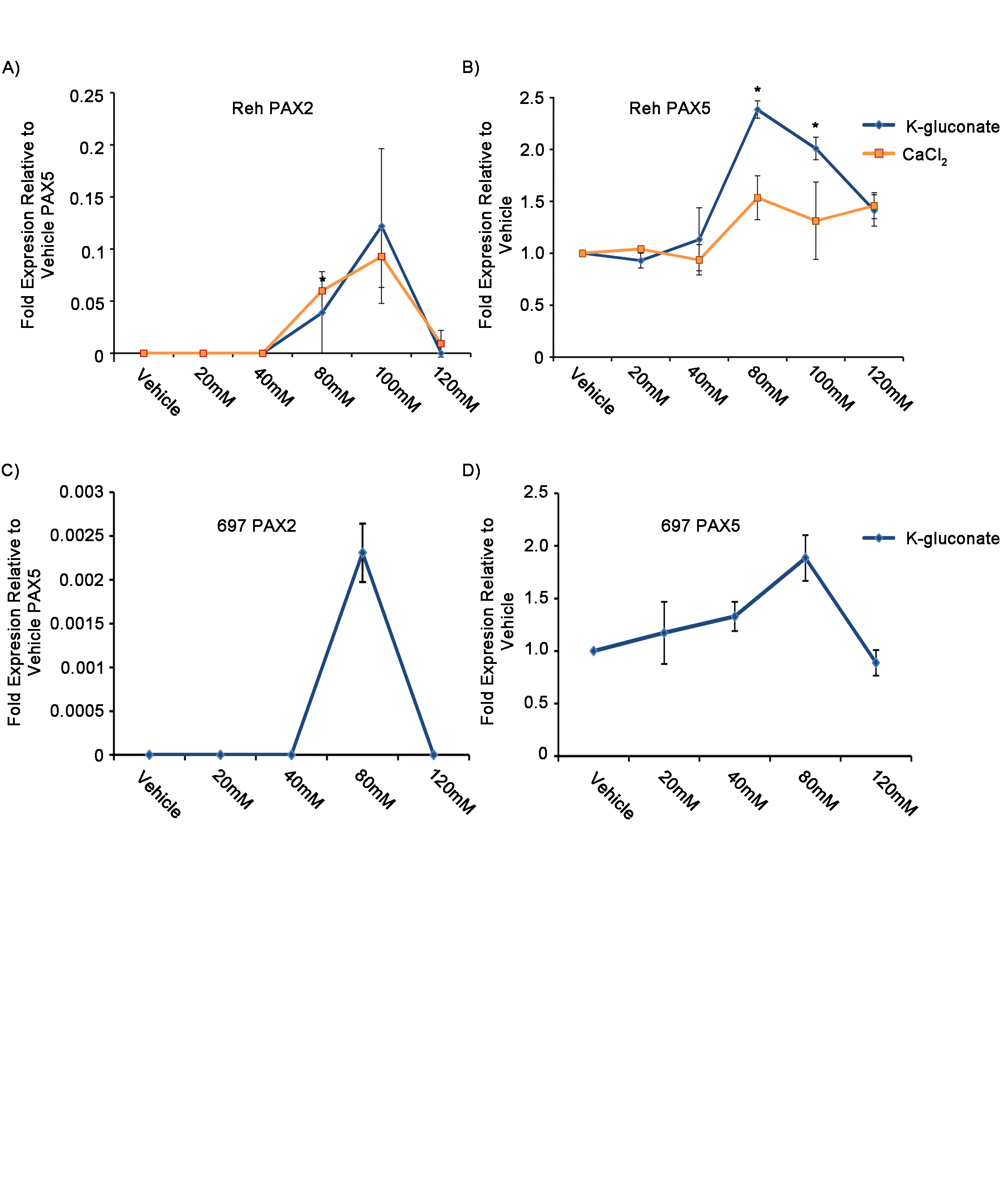

Supplement: S7 Fig — A, B) Dose curve as in Fig 5C and 5D, except normalized to GAPDH rather than ACTB. C, D) Dose curve for K-gluconate treated 697 cells, normalized to ACTB. Note, both A and B represent an average of two experimental replicates. (TIF) [file pgen.1007642.s007.tif]

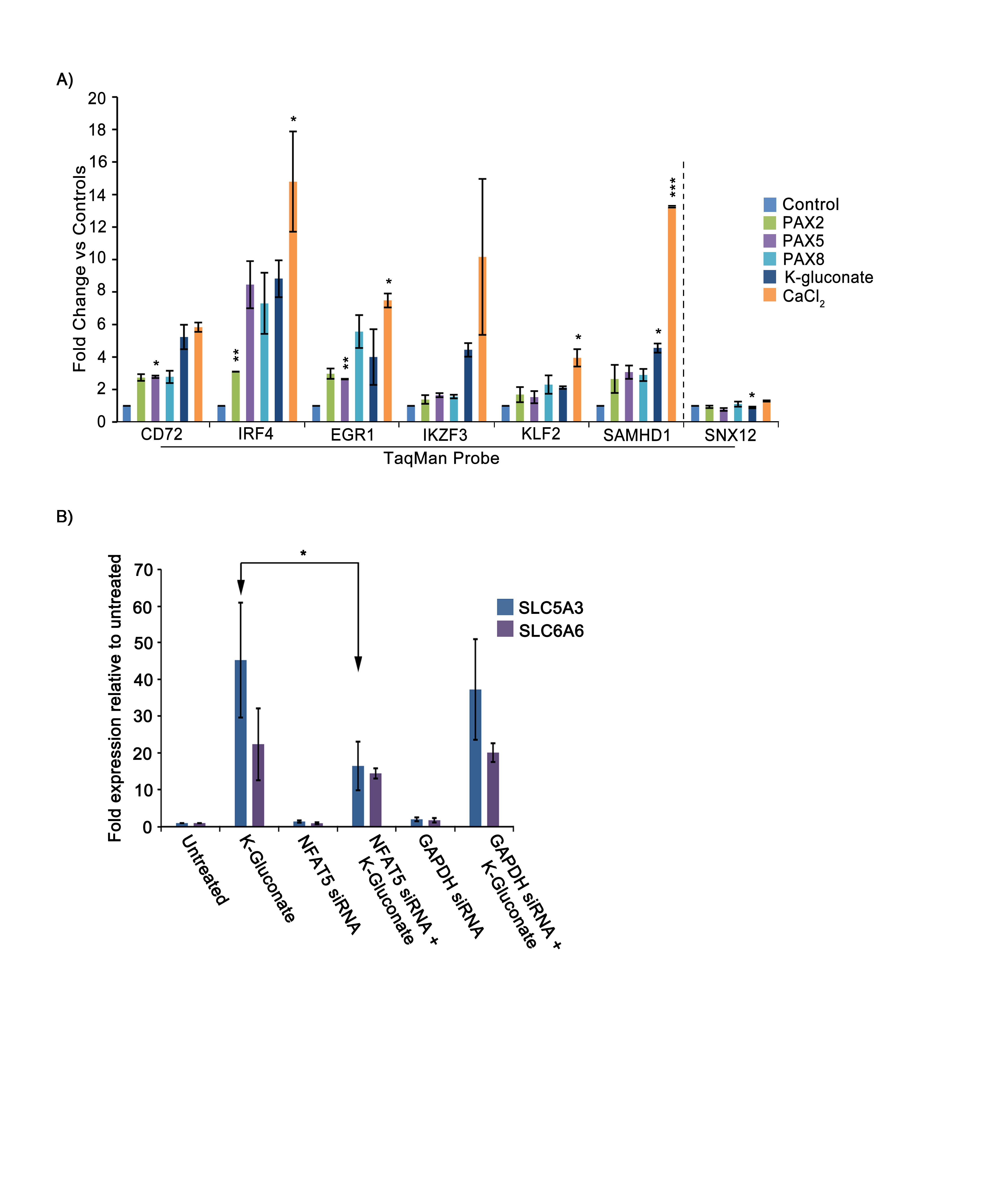

Supplement: S8 Fig — A) qRT-PCR validation of RNA-seq gene subset from Fig 8. Fold change values are 2-ΔΔCT, relative to each samples’ respective control (i.e., empty vector or untreated), with ACTB used as endogenous reference gene. Represents 2 experimental replicates. B) Fold expression of solute channels (+/-) 80mM K-gluconate and (+/-) siRNA knockdown of NFAT5 or GAPDH as a negative control. Represents 3 experimental replicates. (TIF) [file pgen.1007642.s008.tif]

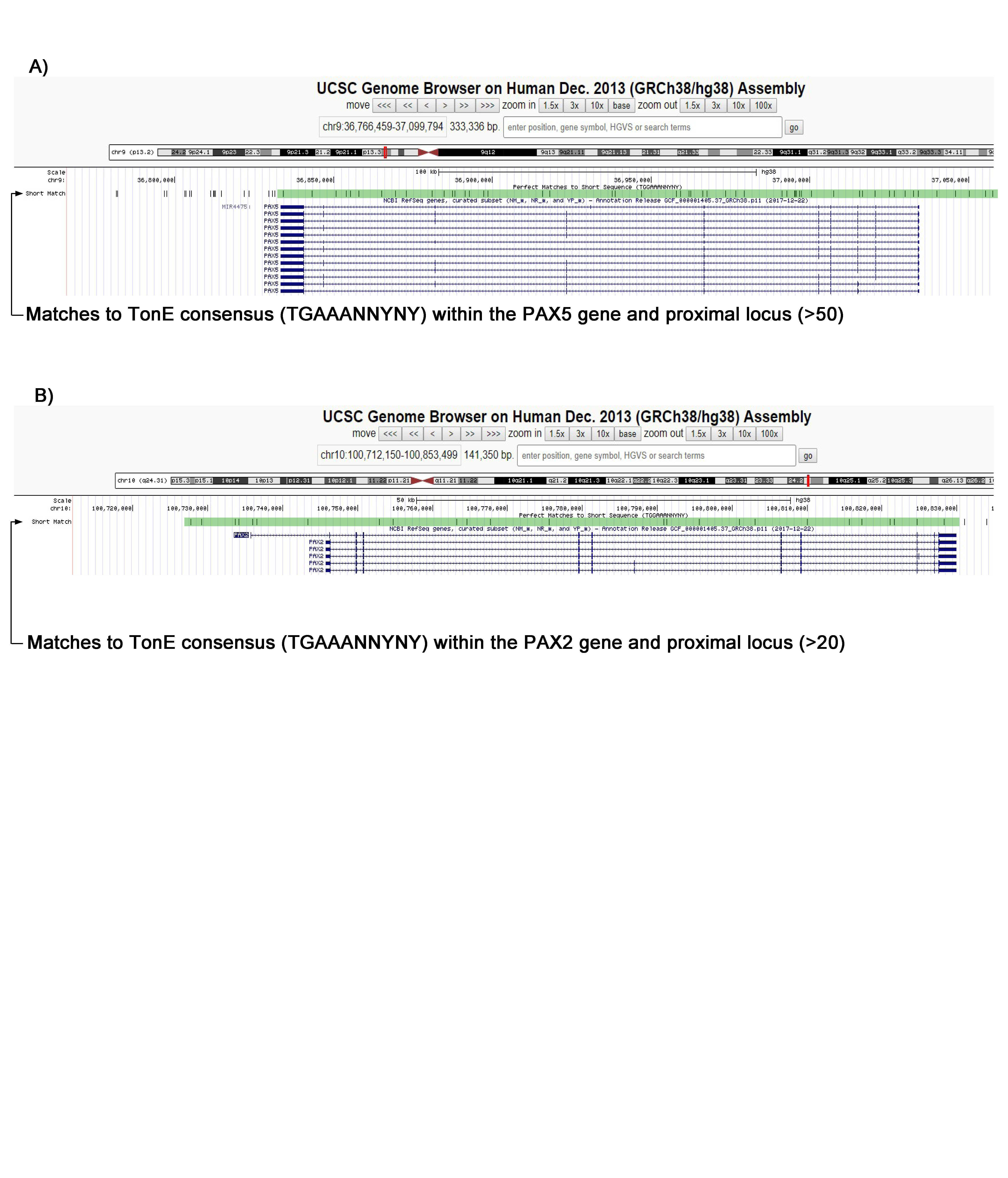

Supplement: S9 Fig — A) Screen shot from UCSC Genome Browser image of the PAX5 locus, highlighting instances of the TonE consensus sequence (TGAAANNYNY) which are present in the genomic region shown. B) As in A, but for the PAX2. (TIF) [file pgen.1007642.s009.tif]

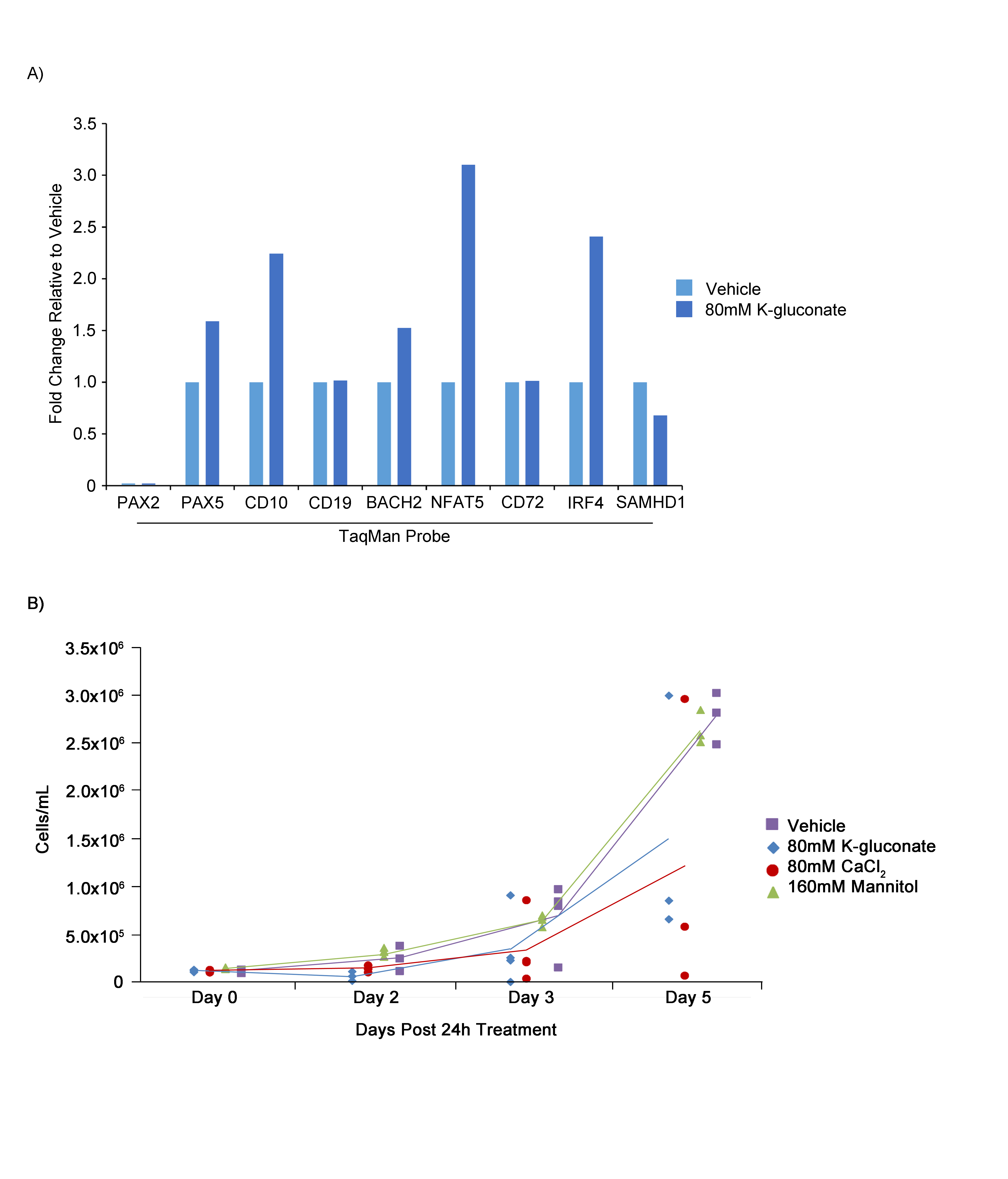

Supplement: S10 Fig — A) qRT-PCR analysis of PAX2, PAX5, and several downstream genes for one aliquot of direct-from-patient, primary sample in response to 24 hour treatment with 80mM K-gluconate. Cells were sorted by FSC-A/SSC-A for live cells prior to isolation/harvest of RNA. B) Reh cells were treated with 80 or 160mM mannitol, 80mM K-gluconate, or vehicle control for 24 hours, followed by FSC-A/SSC-A sorting for 2×10^5^ live cells per condition which were then return to culture. Culture density as shown, was evaluated manually at days 2, 3, and 5 post sort. Data points for 3 experimental replicates are shown, as are lines representing mean values of combined replicates. (TIF) [file pgen.1007642.s010.tif]

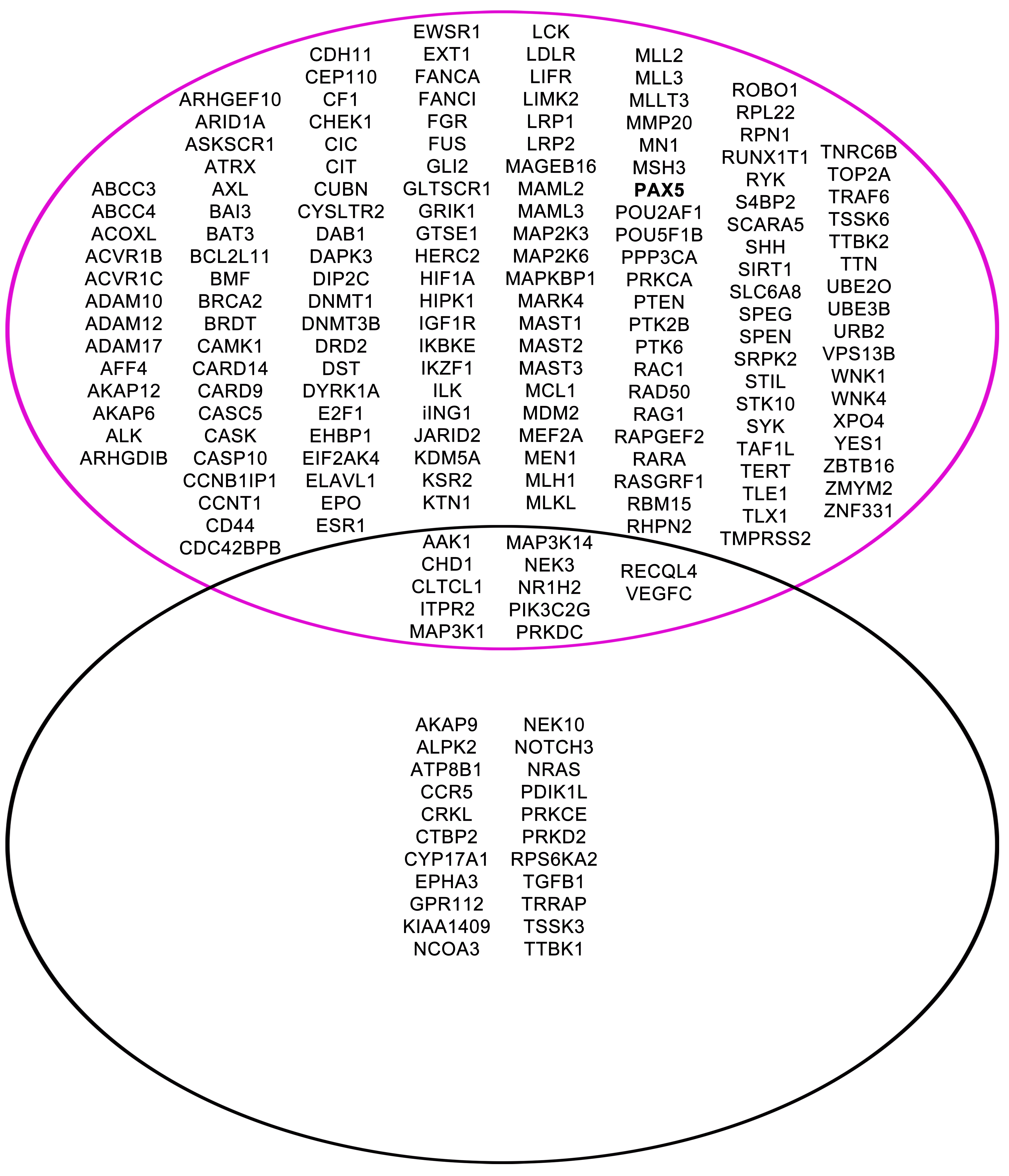

Supplement: S11 Fig — Shown is a Venn diagram of all coding mutations listed in the CCLE for both 697 (black) and Reh cells (magenta). Note, while the CCLE shows a PAX5 mutation in 697 cells, it is not included here as we did not detect that mutation by Sanger sequencing (see S13 Fig). However, we did confirm 697 identity by verifying the presence of other mutations and by short tandem repeat profiling (S14 Fig). Additionally, the p.A322fs PAX5 mutation in Reh cells is not reported by the CCLE, but is shown here (bold), as it has been reported by other sources, and we have verified it by Sanger sequencing (see Methods, S13 Fig). (TIF) [file pgen.1007642.s011.tif]

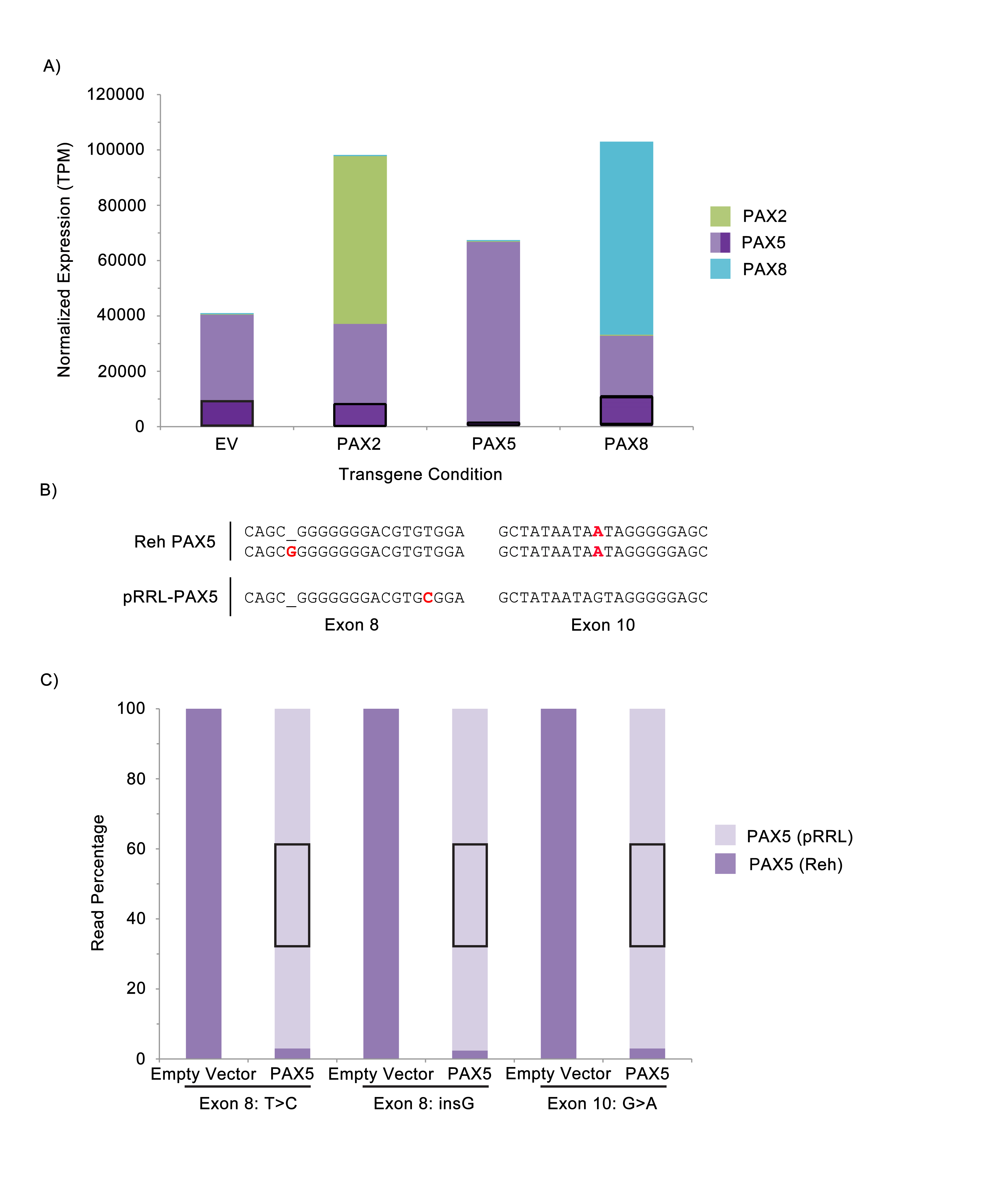

Supplement: S12 Fig — A) Normalized RNA-seq expression data of PAX2/5/8 in transfected Reh cells. rlog normalized TPM values shown in cells transfected with pRRL- empty vector, PAX2, PAX5, or PAX8. Dark purple box indicates proportion of reads from Reh p.A322fs allele. B) PAX5 variant sequences in Reh cells and pRLL-PAX5 cDNA. (+) strand genomic sequence is shown for exon 8 and 10 for both Reh alleles as well as pRLL-PAX5. Variants are shown in red. C) Proportion of PAX5 aligned reads attributable to either the Reh alleles or the pRLL-PAX5. For Exon 8: insG, the ratio of reads with an insertion to total reads in the empty vector sample from A was used to estimate the percentage of reads attributable to the Reh alleles. Black boxes indicate the range of Reh PAX5 expected based on samples in A. Average read depth (SD) for each condition; Empty Vector = 570(65), PAX5 = 11×103 (2.1×10^3^). Exon 8: T>C = NC_000009.12:g.36,882,065T>C, Exon 8: insG = NC_000009.12:g.36,882,053insG, Exon 10: G>A = NC_000009.12:g.36,840,626G>A. (TIF) [file pgen.1007642.s012.tif]

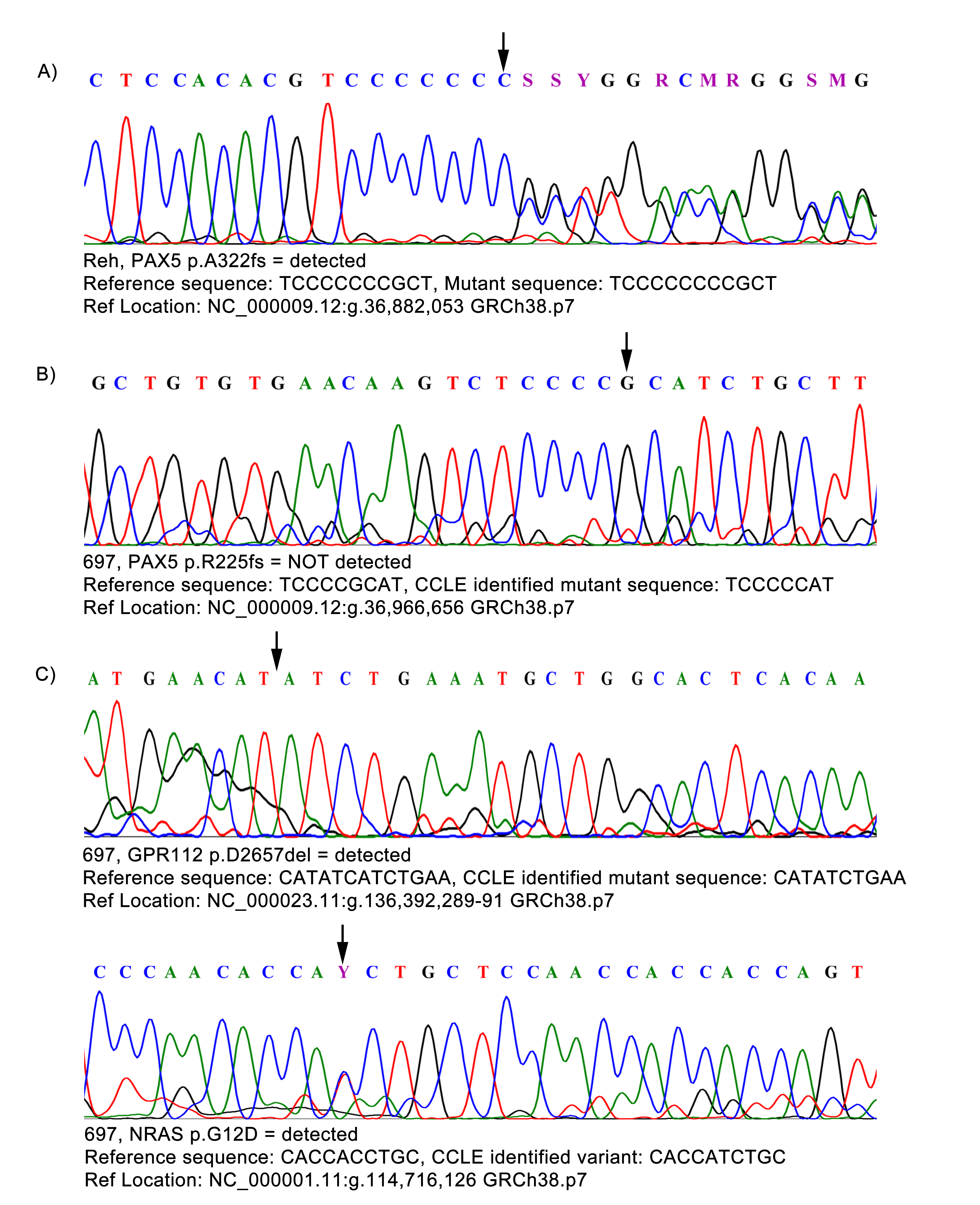

Supplement: S13 Fig — Arrows denote the locations of previously reported mutations for Reh and 697 cells. A) Electropherogram from Sanger DNA sequencing of exon 8, Reh cells, showing a heterozygous single nucleotide insertion resulting in the p.A322fs mutation [33]. B) Electropherogram trace from exon 6, 697 cells, at the location of the CCLE identified p.R225fs mutation [71]. C) Electropherogram traces showing the presence of GPR112 and NRAS mutations (p.D2657del and pG12D) as reported in the CCLE for the 697 cell line. Note that GPR112 resides on the X chromosome, and the cell source is male. (TIF) [file pgen.1007642.s013.tif]

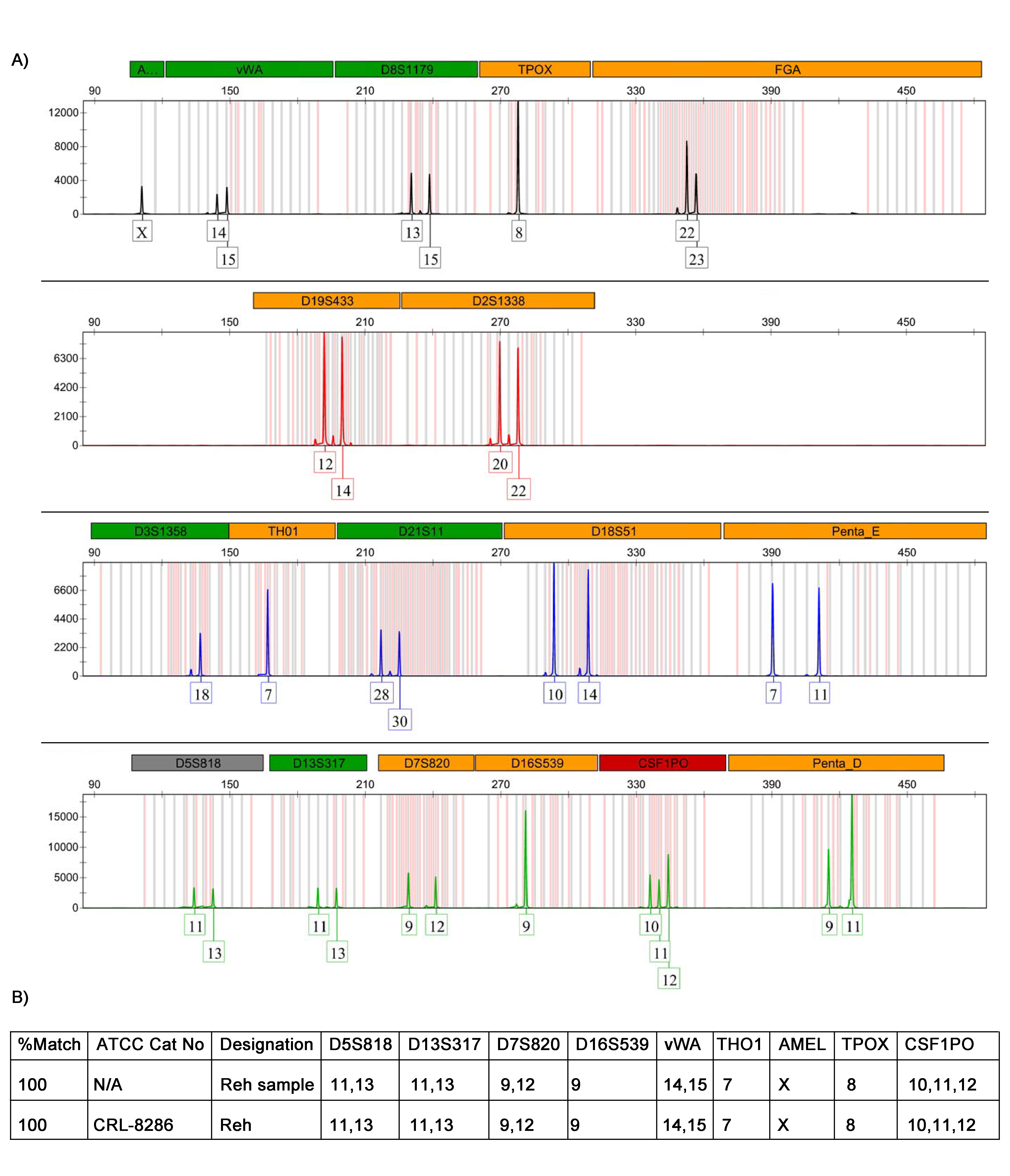

Supplement: S14 Fig — A) Electropherogram results for the Reh sample. B) Profile comparisons for the submitted sample and ATCC CRL-8286 (Reh cell line) for a subset of 18 evaluated loci. (TIF) [file pgen.1007642.s014.tif]

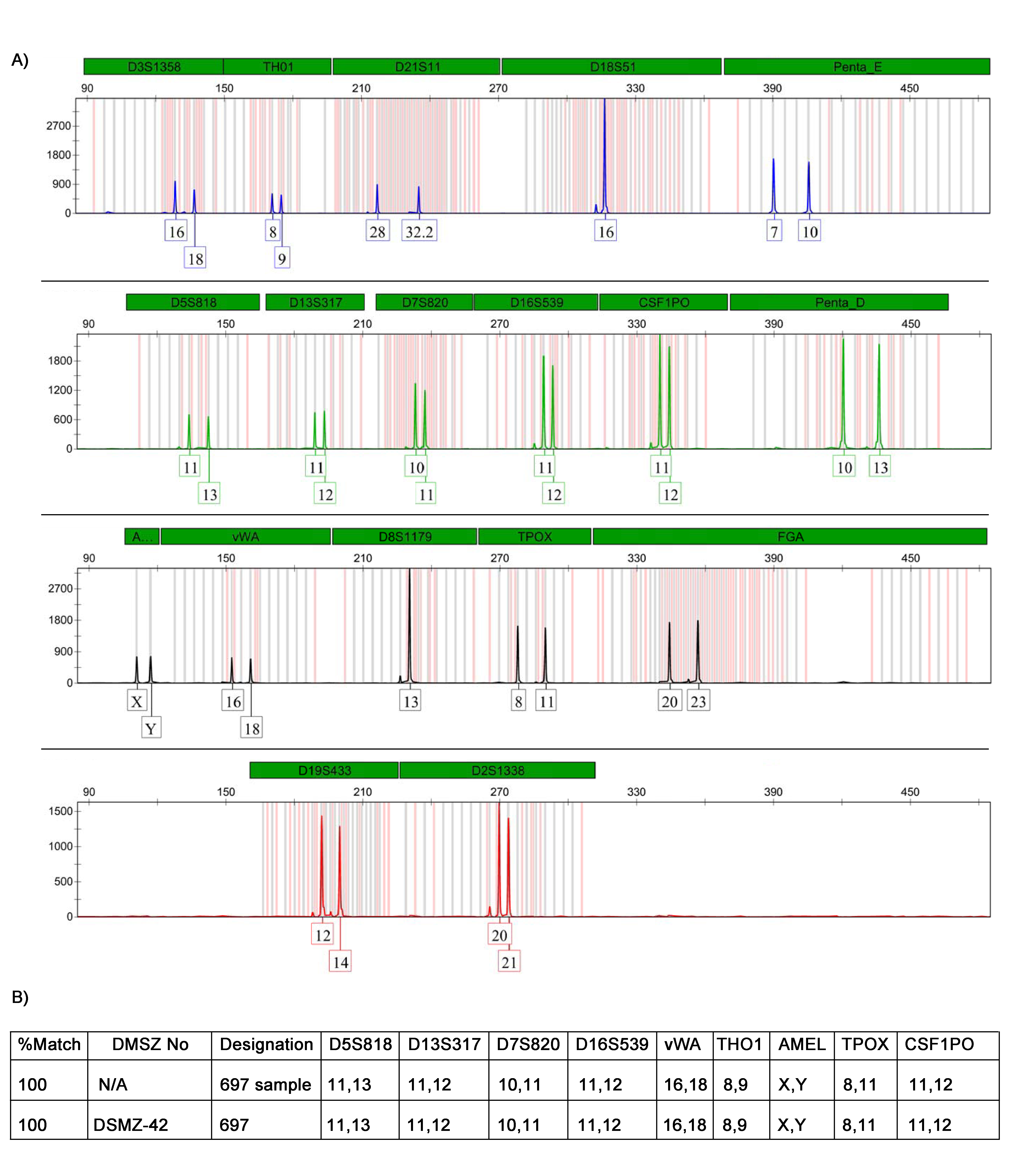

Supplement: S15 Fig — A) Electropherogram results for the 697 sample. B) Profile comparisons for the submitted sample and DSMZ-42 (697 cell line) for a subset of 18 evaluated loci. (TIF) [file pgen.1007642.s015.tif]

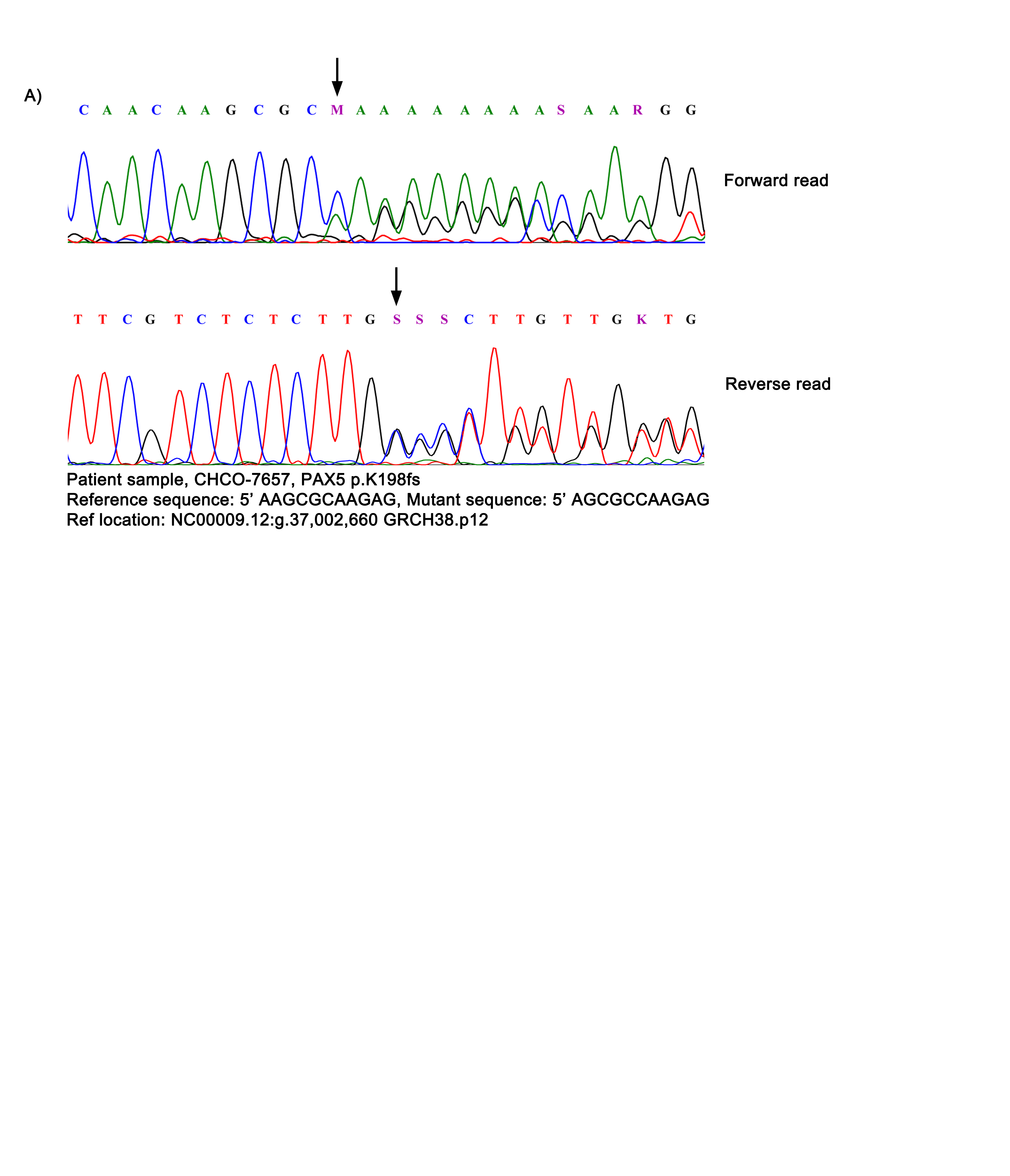

Supplement: S16 Fig — Forward and reverse Sanger traces show position of the p.K198fs mutation in PAX5. (TIF) [file pgen.1007642.s016.tif]
